# Multi‐Material Droplet‐Based Hydrogel Threads for Extrusion 3D Printing

**DOI:** 10.1002/smtd.202500928

**Published:** 2025-11-02

**Authors:** Dor Tillinger, Nicholas X. Armendarez, Joseph S. Najem

**Affiliations:** ^1^ Department of Mechanical Engineering The Pennsylvania State University 336 Reber Building State College PA 16802 USA

**Keywords:** droplet interface bilayers, droplet‐based printing, hydrogels, microfluidics, multi‐material 3D printing, multifunctional materials, oil siphoning

## Abstract

Multi‐material 3D printing holds significant promise for fabricating complex structures, but is hindered by viscosity incompatibility and material cross‐contamination. These limitations stem from the two dominant printing methods: extrusion and inkjet. Extrusion printing enables precise deposition of high‐viscosity materials but suffers from cross‐contamination. In contrast, inkjet printing effectively manages low‐viscosity inks in distinct material compartments, but lacks precision, scalability, and accurate droplet placement. This study introduces a multi‐material hydrogel thread fabrication technique that integrates the strengths of both methods. The threads consist of distinct, aqueous hydrogel droplets generated using a microfluidic chip within an oil stream and brought into contact through a continuous oil siphoning region. Phospholipids in the oil phase prevent droplet fusion while promoting adhesion by forming phospholipid bilayers between neighboring droplets. These assembled threads are then deposited using a 3‐axis stage and cured into stable hydrogel structures. The technique's ability to achieve high‐resolution structures is demonstrated by successfully printing Hilbert curve‐based patterns. This printing approach for soft, multi‐material structures enables precise material deposition, minimizes cross‐contamination, and facilitates effective compartmentalization, thereby bridging the gap between extrusion and inkjet printing. It enables scalable production of complex structures with diverse properties for applications in tissue engineering, soft robotics, and biofabrication.

## Graphical abstract

This study introduces a novel approach to produce multi‐material threads for 3D printing by precisely arranging hydrogel droplets separated by lipid bilayers. By merging the precision of inkjet printing with the scalability of extrusion printing, this method prevents cross‐contamination, maintains material control, and enables intricate designs—unlocking new possibilities for advanced soft robotics, biomedical devices, and tissue engineering applications.

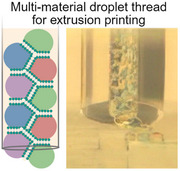

## 1 Introduction

To replicate the functional complexity found in biological systems, engineered materials and systems must emulate the intricate structural and compositional diversity of natural and biological architectures.^[^ ^1^, ^2^, ^3^ ^]^ Multi‐material 3D printing has emerged as a highly effective and promising approach to fabricate such biomimetic systems.^[^ ^2^, ^3^ ^]^ Among various techniques, extrusion‐based printing has evolved from the traditional deposition of single‐material filaments to multi‐material capabilities by integrating multiple reservoirs into a single nozzle or employing multi‐nozzle systems.^[^ ^4^ ^]^ For instance, a system linking seven independent reservoirs to a single nozzle can extrude up to seven distinct inks concurrently in a continuous filament has been demonstrated by Liu et al. ^[^ ^5^ ^]^ In contrast, Skylar‐Scott et al. ^[^ ^6^ ^]^ developed a multi‐material nozzle that can deposit a voxelated filament incorporating up to eight distinct materials. In this setup, the desired material is selected by activating pneumatic solenoids, while the switching frequency controls the quantity deposited among various solenoids. Although these innovations facilitate the creation of complex and patternable structures, they face critical limitations. For example, they usually require high‐viscosity inks, often around the range of 10^7^ mPa s^−1^,^[^ ^7^ ^]^ rendering them unsuitable for the fabrication of hydrogel‐based systems. Moreover, they suffer from cross‐contamination between materials and strict requirements for uniform environmental conditions (e.g., pressure and temperature). Furthermore, incorporating living organisms, such as cells or bacteria, is challenging due to shear stresses imposed by the nozzle walls.^[^ ^4^ ^]^

Alternatively, inkjet printing methods deliver material in discrete droplets with lower viscosity, around 5 − 10 mPa s^−1^, and finer resolution of approximately 50 µm, compared to 100 µm achieved with extrusion methods.^[^ ^4^ ^]^ Inkjet printing also mitigates cross‐contamination, as it uses independent compartments for each material, thereby reducing nozzle obstructions and enhancing its viability for cell‐based bioprinting.^[^ ^4^ ^]^ For instance, Xu et al. demonstrated inkjet co‐printing of human stem cells, canine muscle cells, and bovine endothelial cells onto the same scaffold, with each cell type exhibiting successful in vitro proliferation and viability.^[^ ^8^ ^]^

**Figure 1:**
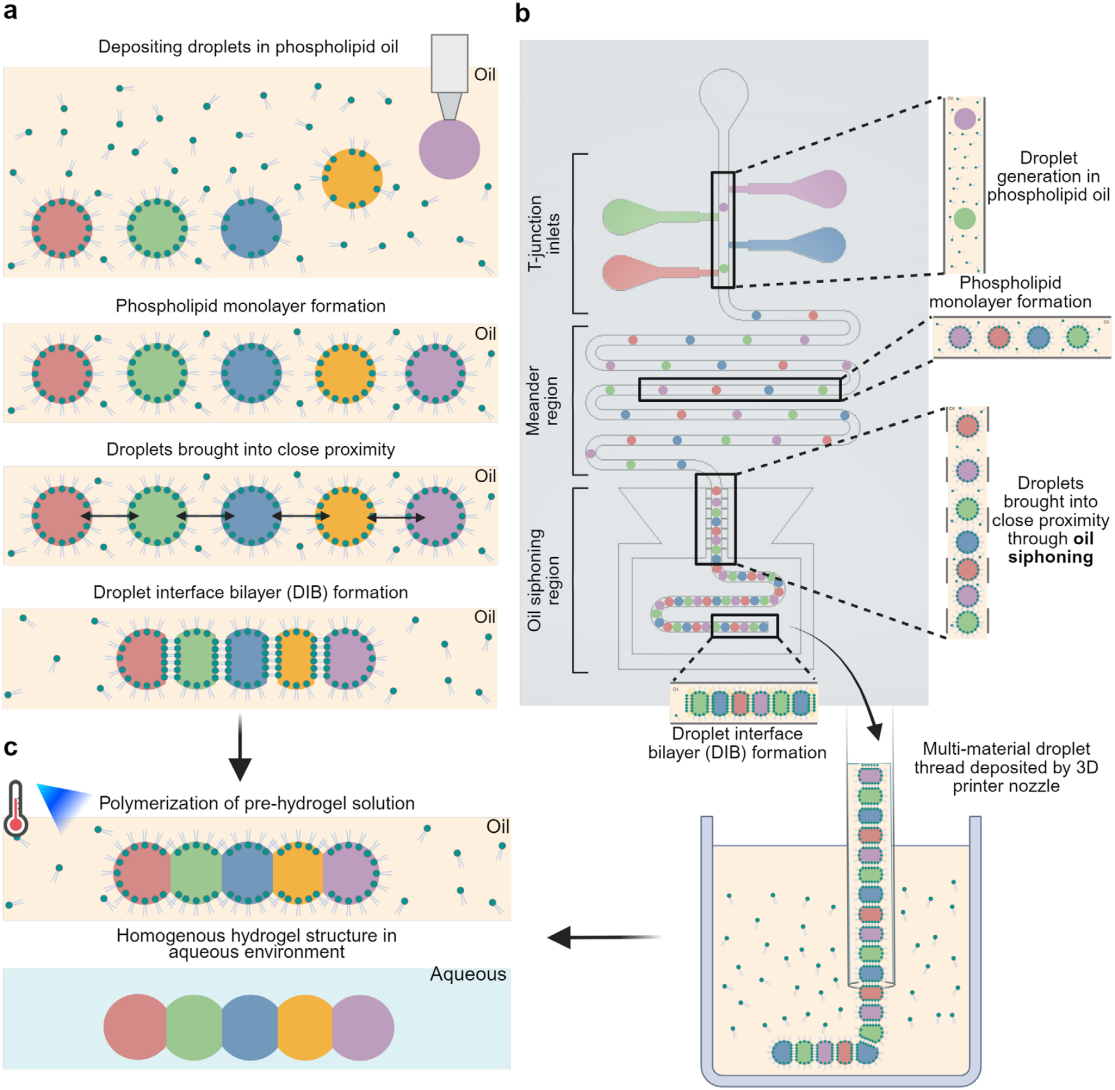
Hydrogel structures assembled from microliter droplets brought together by means of droplet interface bilayers (DIBs). a) A DIB‐assembled droplet network can be manually formed using a pipette or automatically with an inkjet printing nozzle.^[^ ^9^ ^]^ When deposited in a phospholipid‐infused oil solution, the aqueous droplets get encased with lipid monolayers at their surfaces.^[^ ^9^ ^]^ When two or more droplets come into contact, a lipid bilayer forms between them rather than coalescing. b) A continuous DIB‐assembled droplet thread can be formed for multi‐material printing using a microfluidic chip. Initially, aqueous droplets are generated through microfluidic T‐junctions and form monolayers as they travel through the meander region within the chip. Excess oil is then removed as the droplets pass through the siphoning region, causing them to come closer together and touch, forming lipid bilayers. Finally, the assembled droplet thread is printed in a vat containing oil infused with phospholipids. c) Pre‐hydrogel solutions are polymerized to create a continuous multi‐material hydrogel by disrupting the lipid bilayers that connect adjacent droplets. The final homogeneous hydrogel structure is then transferred into an aqueous solution.

To further enhance voxelization and material compartmentalization, inkjet approaches have been combined with droplet interface bilayer (DIB) techniques.^[^ ^9^, ^10^ ^]^ These techniques consist of injecting aqueous droplets into an oil phase containing phospholipids that self‐assemble at the oil–water interface to form lipid monolayers. When droplets come into contact, the monolayers between neighboring droplets form lipid bilayers, creating stabilized and compartmentalized droplet networks (**Figure** **1**a). In particular, DIB‐enabled inkjet printing has been used to print tissues and tissue‐like materials and has displayed some of the highest post‐printing cell viability rates.^[^ ^11^, ^12^, ^13^ ^]^ In addition to its bioprinting successes, the DIB technique is versatile and is gaining traction in various fields, including soft actuators, cell scaffolding matrices, soft power systems, iontronics, sensors, and neuromorphic computing.^[^ ^9^, ^11^, ^13^, ^14^, ^15^, ^16^, ^17^, ^18^, ^19^, ^20^, ^21^, ^22^ ^]^ However, despite its advantages, inkjet printing remains limited by variable droplet sizes, reduced deposition precision, and low mechanical strength of pre‐polymerized inks, hindering the fabrication of complex, clinically relevant structures.^[^ ^4^, ^23^ ^]^

To address these limitations, we introduce a multi‐material hydrogel droplet thread printing platform that integrates the scalability and structural complexity of extrusion printing with the low‐viscosity ink compatibility and reduced cross‐contamination of inkjet methods. The process involves producing a continuous droplet thread within a microfluidic chip, which is then extruded into an oil vat for 3D printing of complex architectures (Figure 1b). Each material stream is introduced through a T‐junction, a standard microfluidic component known for reliably generating monodisperse droplets.^[^ ^24^, ^25^, ^26^ ^]^ The droplets then pass through a meander region, a serpentine channel that provides sufficient time for phospholipids to assemble into dense monolayers at the droplet surfaces. An insufficient meander length may result in incomplete monolayer formation and droplet coalescence during bilayer formation. Downstream, the droplets enter a siphoning region where they come into close contact, allowing the formation of lipid bilayers and the assembly of the droplet thread before extrusion into the printing vat (Figure 1b). After printing, the structures are cured into solid multi‐material hydrogel constructs (Figure 1c).

A key innovation of our platform is the dynamic siphoning strategy for continuous droplet assembly. Although previous studies explored microfluidic siphoning primarily for transient droplet manipulation,^[^ ^27^, ^28^, ^29^ ^]^ our method enables sustained droplet thread formation over extended durations, providing a robust and scalable solution for multi‐material hydrogel printing.

## 2 Results and Discussion

### 2.1 Droplet Generation and Monolayer Formation

To automate the creation of the multi‐material droplet thread, we fabricated a microfluidic chip (Section “Fabrication of Microfluidic Chip”) to generate droplets and adhere them together using phospholipid bilayers. Droplet generation is achieved through T‐junction inlets, each connected to a reservoir containing a specific hydrogel precursor solution (Figure 1b, see Figure S1, Supporting Information, for T‐junction dimensions). T‐junctions are commonly used to generate droplets where the two immiscible fluids flowing in orthogonal channels at controlled flow rates intersect.^[^ ^30^, ^31^ ^]^ The continuous phase, which usually flows at a higher flow rate, shears the dispersed phase as it enters the main channel, breaking it into uniform monodisperse droplets.^[^ ^30^ ^]^ T‐junction designs are favored due to their simplicity and ability to reliably produce droplets with consistent size distributions.^[^ ^30^, ^31^ ^]^

In our system, a continuous phase consisting of 2 mg mL^−1^ of 1,2‐diphytanoyl‐sn‐glycero‐3‐phosphocholine (DPhPC) phospholipids dissolved in a 1:1 oil mixture of undecane and silicone oil (hereafter referred to as “lipid oil”) flows through the main channel. Various aqueous acrylamide‐based hydrogel solutions (Section 4) are introduced as dispersed phases through the T‐junction inlets. As the generated droplets flow through the main channel, the phospholipids present in the oil phase encase them with a monolayer, preventing coalescence and promoting adhesion between neighboring droplets through lipid bilayers.^[^ ^9^, ^10^ ^]^ Each aqueous solution is supplied through its dedicated T‐junction inlet, which intersects the main channel through which the lipid oil flows (Figure 1b). Three such T‐junction inlets are placed at alternating intervals of 1.1 mm along the main channel, enabling the periodic generation of three distinct hydrogel droplet types through pressure‐driven flow (see Figure S1 for T‐junction dimensions and Movie S1, Supporting Information). The choice of three inlets is based on the available input channels in our pressure controller (see Experimental Section 4). However, our microfluidic design is scalable, allowing for additional inlets to accommodate more hydrogel types. Furthermore, solutions with similar viscosities can be connected to the same pressure channel, enabling expansion without extra equipment. When increasing the number of inlets, the spacing between the alternating T‐junction inlets may need adjustment to maintain proper droplet sequencing. In our configuration, droplet generation rates are synchronized at ≈2 Hz, ensuring that droplets from each inlet enter the main channel in a defined sequence. The 1.1 mm spacing between T‐junction ensures that the droplet order remains constant, with the first droplet in the sequence entering from the T‐junction inlet closest to the meander region and the last droplet in the sequence entering from the T‐junction inlet farthest from the meander region (Figure 1b). The distance between the T‐junction inlets provides sufficient time for all generated droplets to enter the meander region without fusing or coalescing with the succeeding incoming droplets being generated (Movie S1, Supporting Information).

The T‐junctions produced droplets with an average volume of 52.6 ± 4.9 nL, corresponding to a mean diameter of 465±15 µm (Figure S2, Supporting Information). These droplet volumes were chosen to demonstrate the capability of 3D printing low‐viscosity inks with larger droplet resolution compared to traditional inkjet printers. Droplet volume is controlled through the difference in flow rate between the continuous phase, in this case the lipid oil, and the disperse phase, the various aqueous hydrogel types.^[^ ^32^, ^33^ ^]^ For a microfluidic system with a laminar flow, the flow rate can be determined by the following relationship:

(1) $$\Delta P_{hyd}=QR_{hyd}$$

where Δ*P*_*hyd*_ is the hydraulic pressure difference between the input pressure and outlet pressure, *Q* is the fluid flow rate, and *R*_*hyd*_ is the hydraulic resistance.^[^ ^32^, ^33^ ^]^ For a rectangular microfluidic geometry, as is employed in the design of this chip and in which the width is much greater than the depth, the hydraulic resistance is commonly defined as:

(2) $$R_{hyd}=\frac{12\mu L}{h^{3}w}\frac{1}{1-0.63\frac{h}{w}}$$

where μ is the fluid viscosity, *L* is the length of the channel, *h* is the height of the channel, and *w* is the width of the channel.^[^ ^32^, ^33^ ^]^ In a laminar flow regime, as in our case, the microfluidic chip can analogously be represented as an electrical circuit (as described in more detail in Section S3, Supporting Information). In this study, Equations (1) and (2) are used throughout the microfluidic chip design. Initially, we used Equations (1) and (2) to determine the flow rates required to generate droplets with our desired volume based on our selected input pressures and geometrical parameters. Furthermore, we used these equations to calculate the length of the meander region, a long continuous channel that provides sufficient time for monolayer formation (Figure 1b). In our chip, the meander region is 88.5 mm in length, which provides ≈30–35 s for a phospholipid monolayer to self‐assemble around the entire surface of each droplet.^[^ ^34^, ^35^, ^36^, ^37^ ^]^ Finally, Equations (1) and (2) govern the geometric dimensions of the siphoning region, a convergence region where the droplets are brought into close contact, allowing lipid bilayers to form at their interfaces and enabling the assembly of the multi‐material droplet thread (Figure 1b).

### 2.2 Theory Governing the Design of Microfluidic Oil Siphoning

**Figure 2:**
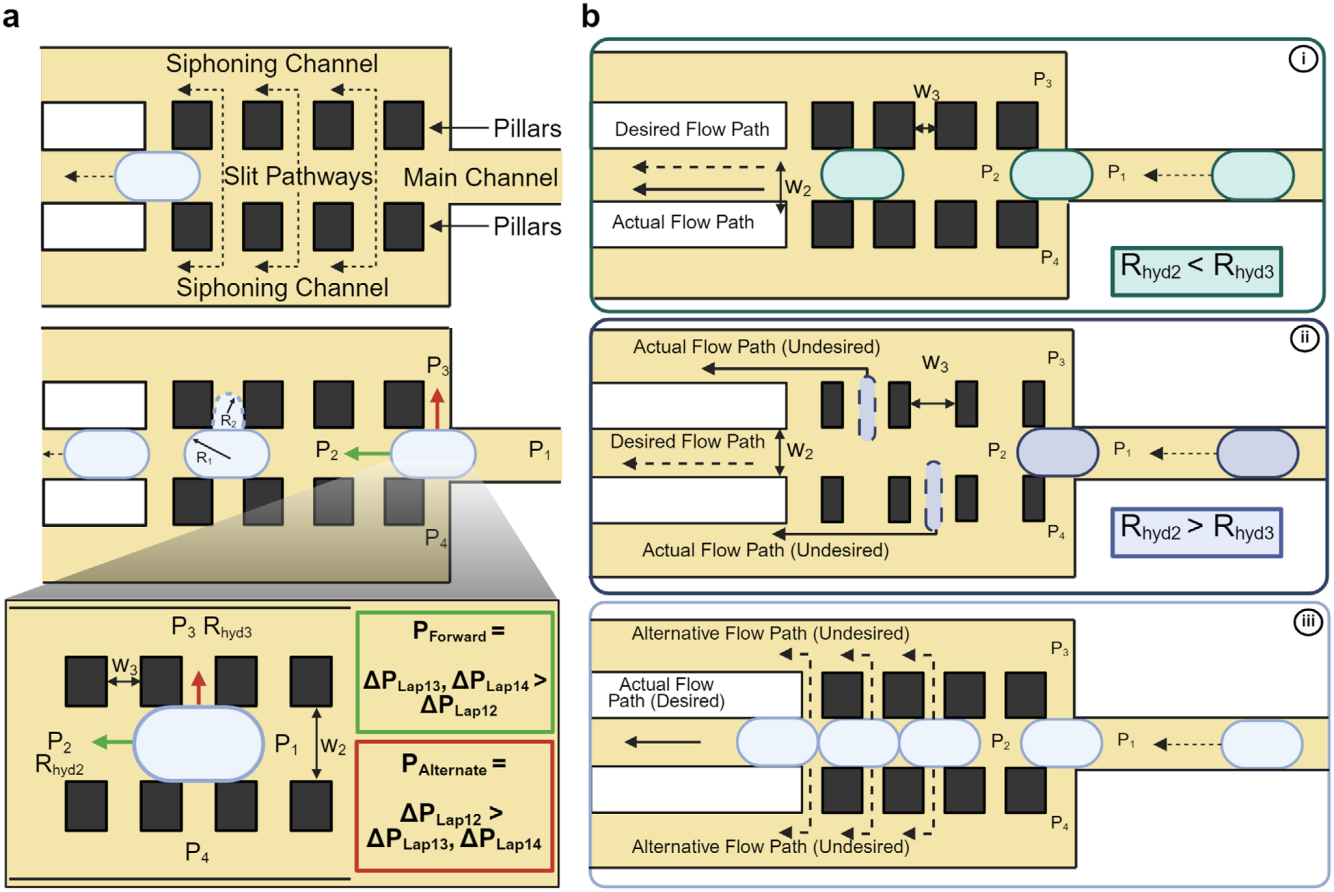
a) Illustration of a droplet in the siphoning region. The siphoning region is created by separating the main channel and the parallel siphoning channels through a linear array of pillars that form small slit pathways. The hydraulic resistance of the slit pathways is large due to their small dimensions, preventing the droplets from deforming while enabling the oil phase to flow into the parallel siphoning channels. The slit pathway dimensions are derived from the Laplace deformation pressure (Equation 3) and indicate the likelihood of the droplet deforming through the slit pathway. The droplet radii of curvature, R_1_ and R_2_, are used to determine the Laplace pressure gradient of the droplet as it interfaces with the slit pathways. The Laplace pressure difference between points P_1_ and P_2_, denoted as ΔP_Lap12_, governs the droplet's forward trajectory. If ΔP_Lap12_ is greater than the pressure differences between points P_1_ and P_3_ or P_1_ and P_4_ (ΔP_Lap13_ and ΔP_Lap14_, respectively), the droplet proceeds along the main channel. Conversely, if ΔP_Lap13_ or ΔP_Lap14_ exceeds ΔP_Lap12_, the droplet will deform and divert into the slit pathways. b) Hydraulic resistance, determined by the geometric design of the siphoning region, guides the flow path of the droplets. i) The resistance to entering the siphoning channel is greater than the resistance of the main channel, promoting movement through the desired pathway. ii) The resistance to enter the siphoning channel is less than that of the main channel, droplets will deform and pass through the slit pathways into the undesired siphoning channels. iii) As multiple droplets accumulate in the siphoning region, the increased downstream pressure raises the likelihood of deformation into the siphoning channels. To maintain continuous droplet flow along the main path, the input hydraulic pressure must be sufficiently increased to overcome the rising hydraulic resistance due to droplet accumulation.

Once generated, the droplets travel through the meander region at a consistent spacing (1.95±0.08 mm, Figure S3, Supporting Information). To bring them into contact for lipid bilayer formation (i.e., adhesion) and ultimately to form the thread, the continuous oil phase must be extracted from the main channel. This extraction reduces the flow velocity and the spacing between droplets, promoting bilayer formation. This can be achieved by adding a siphoning region at the end of the meander channel. A siphoning region is a section in the microfluidic chip in which only the continuous oil phase is extracted, while the droplets, the disperse phase, continue to travel along the main channel.^[^ ^27^, ^28^, ^29^, ^36^, ^38^ ^]^ A microfluidic siphoning region is traditionally constructed by having a siphoning channel in parallel to the main channel separated by pillars with small gaps in between them that create narrow slit pathways (**Figure** **2**a).^[^ ^27^, ^28^, ^29^, ^36^, ^38^ ^]^ The dimensions of the slit pathways are small to allow the oil phase to flow into the siphoning channel while preventing the droplets from entering the slit pathways, as the hydraulic resistance around the slit pathway is greater than that of the main channel.^[^ ^27^, ^28^, ^29^, ^36^, ^38^ ^]^ This method has previously been used in assay collections and droplet coalescence studies.^[^ ^27^, ^28^ ^]^ Additionally, the droplet interface bilayer (DIB) method has previously been integrated with a siphoning region to capture droplets and control the droplets' configuration and bilayer contact areas.^[^ ^29^, ^36^, ^38^ ^]^ In this paper, we use the same DIB method to adhere the droplets into threads through the formation of lipid bilayers. However, in these previous methods, the siphoning regions are activated only to bring droplets closer together or to manipulate droplet configurations after bilayer formation is complete and stable.^[^ ^29^, ^36^, ^38^ ^]^ The actual formation of the lipid bilayer occurs statically when the siphoning region is inactive.^[^ ^29^, ^36^, ^38^ ^]^ In our case, when continually constructing a droplet thread, the lipid bilayer must form dynamically while the droplets are in transit.

The design of a continuous siphoning region must prevent droplets from escaping, either partially or fully, through the slit pathways, as this could disrupt the sequence and size of the droplets. The Young–Laplace equation or the Laplace deformation pressure (Equation 3), which describes the pressure required to deform a droplet as it passes alongside a slit pathway, is used to determine the appropriate dimensions of the slit pathways.^[^ ^27^, ^39^ ^]^ The Laplace deformation pressure of droplets confined within microfluidic geometries can be calculated as follows:

(3) $$\Delta P_{Lap}=\gamma \left(\frac{1}{R_{1}}+\frac{1}{R_{2}}\right)$$

where Δ*P*_*Lap*_ represents the pressure difference across the anterior interface of the droplet and the posterior interface of the droplet, γ denotes the interfacial tension between the droplet and the lipid oil, and *R*_1_ and *R*_2_ refer to the radii of curvature of the anterior and posterior interfaces of the droplet as they interface different channels and pathways, respectively, (Figure 2a).^[^ ^27^, ^39^, ^40^ ^]^ When the droplets are smaller than the channel dimensions, they will assume a spherical shape. In this case, the droplets' radii of curvature,*R*_1_ and *R*_2_, are equal, which simplifies the Laplace equation to the general Young–Laplace equation as follows:^[^ ^39^, ^40^ ^]^

(4) $$\Delta P_{Lap}=\frac{2\gamma }{R}.$$

A comparison of the Laplace deformation pressure gradients acting on a droplet within the siphoning region helped us determine whether the droplet will deform through the slit pathway and enter the siphoning channel or remain in the main channel, which is the desired outcome (Figure 2a). When a droplet is located in the siphoning region, the hydraulic pressure gradient (calculated by Equation 1) between points P_1_ and P_2_ is the primary force driving it forward along the main channel (indicated by green arrow in Figure 2a). However, when the droplet encounters the slit pathways, additional pressure gradients arise between the points P_1_ and P_3_, and between P_1_ and P_4_, which also act on the droplet (indicated by red arrow in Figure 2a). To ensure that the droplet continues to move along the main channel without deforming into the slit pathways, the Laplace pressure gradients between points P_1_ and P_3_, and between P_1_ and P_4_, must be greater than the Laplace pressure gradient between points P_1_ and P_2_ (Zoomed in view of Figure 2a). This can be achieved by geometrically designing the siphoning region such that the main channel width, w_2_, is larger than the width of the slit pathway w_3_. Consequently, based on Equation (2), the hydraulic resistance along the main channel, R_hyd2_, will be lower than the hydraulic resistance required to enter the siphoning channel through the slit pathway R_hyd3_ (Figure 2b–i). However, if the width of the slit pathway is not sufficiently smaller than the main channel width, the hydraulic resistance across the slit pathways decreases. The Laplace deformation pressure gradient between points P_1_ and P_3_ or between P_1_ and P_4_ reduces below that between points P_1_ and P_2_. This results in the droplet deforming through the slits and entering the siphoning channel, which is undesirable (Figure 2b–ii).

During continuous siphoning operations, droplets accumulate along the main channel in the siphoning region. This aggregation increases the downstream pressure at point P_2_, consequently increasing the Laplace deformation pressure gradient between P_1_ and P_2_. As more droplets accumulate, the likelihood of droplet deformation through the slit pathways also rises. The input hydraulic pressure must be sufficiently high to ensure that the droplet thread continues to advance without droplets deforming into the parallel siphoning channels. This condition requires that the Laplace deformation pressure between points P_1_ and P_2_ remains lower than that between P_1_ and P_3_ or P_1_ and P_4_ (Figure 2b‐iii). The most effective method to control these pressure gradients across various slit pathways is to regulate the oil extraction rate by designing the geometric dimensions of the siphoning channels in relation to the input pressures.

### 2.3 Control of Oil Siphoning Rate for Continuous Droplet Thread Formation

Controlling the oil siphoning rate is critical for successfully assembling a continuous droplet thread. Proper siphoning enables droplets to touch and form lipid bilayers for adhesion and minimizes droplet deformation and coalescence. The most effective way to modulate the oil siphoning rate is by adjusting the dimensions of the siphoning channel within the microfluidic chip. Changes in these dimensions alter the hydraulic resistance of the channel, which in turn affects the flow rate for a given pressure gradient, as described in Equations (1) and (2).

**Figure 3:**
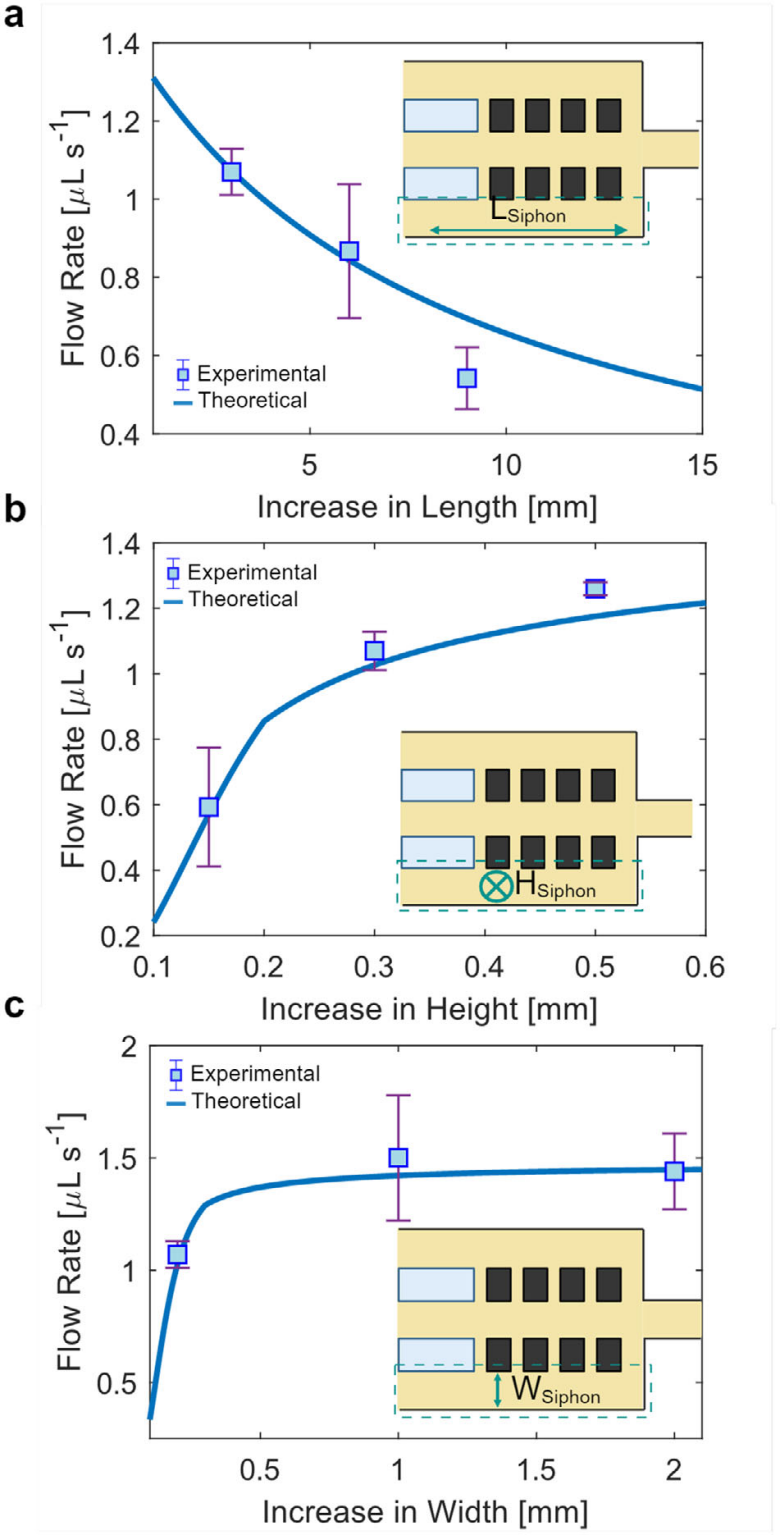
a) The siphoned oil flow rate decreases exponentially with increasing length of the siphoning region. Although the number of slit pathways increase with channel length to accommodate the extended region, their effect on the flow rate is minimal, as confirmed in Figure S7 (Supporting Information). b) Increasing the siphoning channel's height to match the main channel's height causes the oil flow rate to increase, similar to an exponential plateau model. The growth rate is steeper when the height dimension is less than the width dimension and shallower when the height dimension is larger than the width dimension. An inflection point occurs when the height and width dimensions are equal. c) Likewise, increasing the width of the siphoning channel also causes the oil flow rate to increase, akin to an exponential plateau model. Similarly to the observation in (b), there is a steeper growth when the width dimension is smaller than the height dimension and a shallower growth when the width dimension is larger. The inflection point occurs when the width and height dimensions match. We determined the experimental results by calculating the difference in flow rates when the siphoning region was cycled from inactive to active (*n* = 3 cycles).

Using an analogous microfluidic circuit model, we estimate the changes in flow rates between the siphoning and main channels based on their relative hydraulic resistances (Figure S4, Supporting Information). Specifically, increasing the length of the siphoning channel leads to an exponential decrease in the oil extraction rate from the main channel (**Figure** **3**a). This is because the hydraulic resistance increases proportionally with channel length (Equation 2). As the length of the main and siphoning channels increases, additional slit pathways are formed as more pillars are added to prevent droplets from entering the siphoning channels. We simulated the impact on the flow rate of the siphoned oil when the number of slit pathways increases for a fixed channel length (Section S5, Supporting Information). As additional slit pathways are added the hydraulic resistance is calculated for the total number of slits. This is repeated until the total number of slit pathways fits within the fixed channel length. For forming continuous droplet threads with average droplet diameters of 465 ± 15 µm, the slit pathway dimensions with the width of 50 µm, length of 300 µm, and depths of 300 µm are effective in minimizing droplet deformation (Figure S1, Supporting Information). With these dimensions, we observed that there is a near‐constant oil extracted flow rate as the number of slit pathways increases (Figure S7, Supporting Information). This simulation was performed for the longest tested siphoning region length of 9 mm as that would have the maximum number of slit pathways. Although each slit pathway has a high individual hydraulic resistance due to its small cross‐section, the slit pathways are arranged in parallel (Figure S4, Supporting Information). This configuration reduces the net resistance, maintaining a relatively stable oil flow rate between the siphoning and main channels.

For both the height and width of the siphoning channel, the oil siphon flow rate exhibits two distinct regimes (Figure 3b,c). At smaller channel dimensions, the flow rate increases rapidly, followed by a gradual rise toward an asymptotic value as the dimensions become larger. This behavior reflects the changing hydraulic resistance, which depends on the aspect ratio of the channel cross‐section. The commonly used hydraulic resistance equation (Equation 2) assumes that the width is greater than the height. However, when the width and height become comparable, a more generalized resistance equation—discussed in Section S4 (Supporting Information)—is required. The observed inflection points in the flow rate curves correspond to conditions where the width and height are equal.

Based on these relationships, we experimentally quantified the changes in flow rate as a function of siphoning channel length, height, and width. Additionally, we evaluated the changes in flow rate among multiple microfluidic chips with the same siphoning channel dimensions to demonstrate consistency and reproducibility among various devices (Figure S8, Supporting Information). We determined that a flow rate of around 1.3 µL s^−1^ is optimal for bringing the droplets into contact to allow adhesion through the formation of the lipid bilayers. Among the geometric parameters, the channel width dimension achieves the required flow rate starting with a minimum channel width of around 0.5 mm (Figure S5, Supporting Information). Although increasing the siphoning channel height from 0.9 mm can theoretically achieve similar flow rates (Figure S6, Supporting Information), we limited the siphoning channel height to being equal to or smaller than the height of the main channel (0.5 mm) to prevent potential back pressure from occurring. Back pressure can arise from pressure imbalances caused by complex geometries or external downstream pressures.^[^ ^41^, ^42^ ^]^ In our setup, the outlet tubing for both the main and siphoning channels feeds into a shared oil reservoir slightly above atmospheric pressure (Figure S15, Supporting Information). Over time, as oil accumulates in the reservoir during printing, the resulting pressure increase can become significant enough to induce back pressure.

### 2.4 Printing of Multi‐Material Droplet Threads

**Figure 4:**
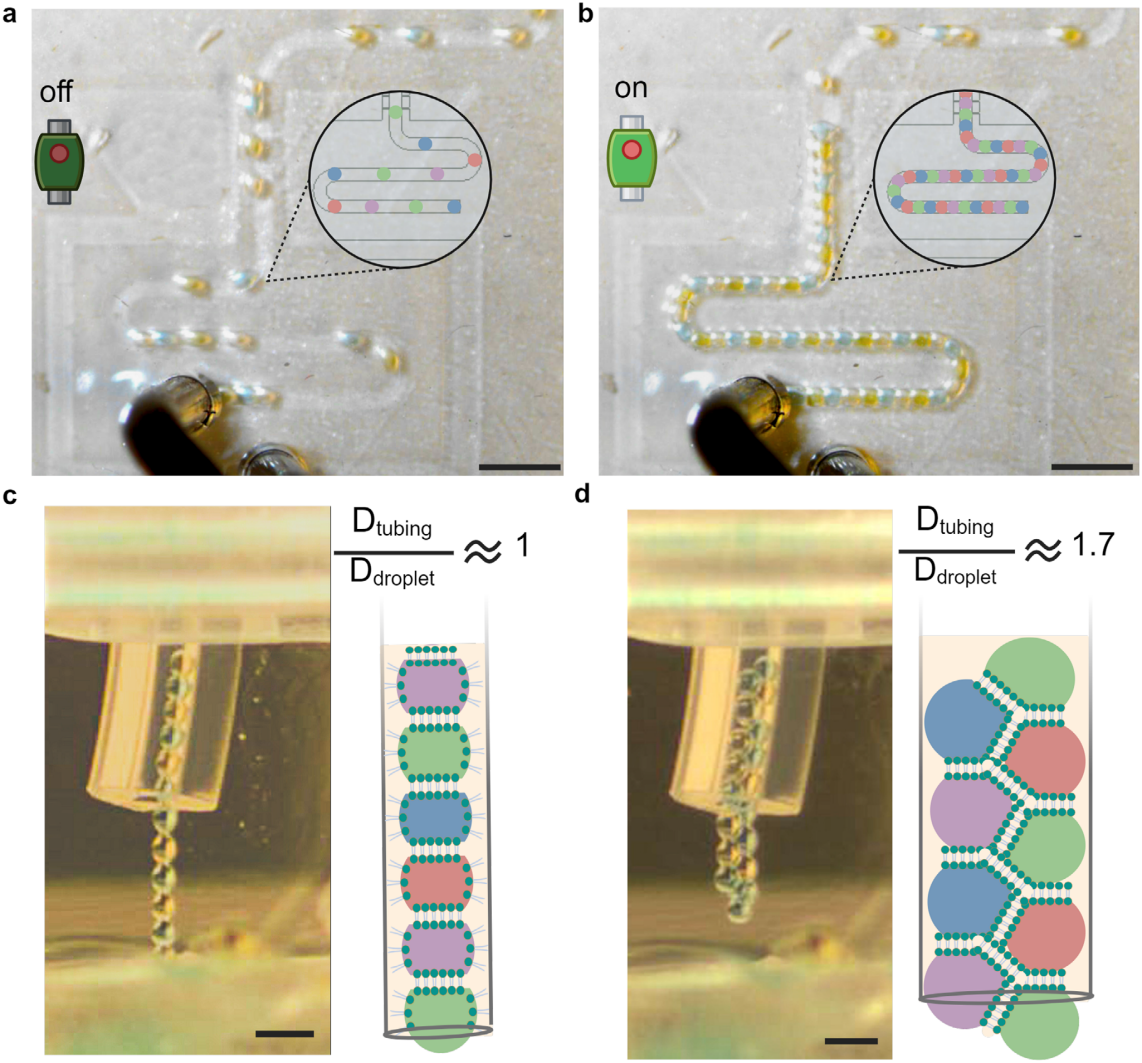
Illustrating the process and outcomes of oil siphoning and hydrogel thread formation. a) When the normally‐closed solenoid valve is off, oil siphoning does not occur, allowing droplets to travel spaced apart along the main channel. b) Oil siphoning occurs when the solenoid valve is turned on, causing oil to flow into the siphoning channels, which reduces the droplets' velocity and proximity until they come into contact and form DIBs (Scale bar = 2 mm for (a) and (b)). c) A single hydrogel thread is produced when the inner diameter of the tubing closely matches the diameter of the droplet. d) The arrangement of the droplet thread varies when the tubing inner diameter exceeds the droplet diameter. The packing configuration will depend on the difference between the tubing and droplet diameters (Scale bar = 1 mm for c and d). The image contrast was adjusted for enhanced visualization without altering the original data.

To demonstrate the capabilities of a continuous, dynamic siphoning region in forming stable, multi‐material, droplet‐based hydrogel threads for 3D printing, we used a three‐axis stage to print a third‐order Hilbert curve. The final siphoning region design and dimensions are detailed in Section S1 (Supporting Information). The primary design change was adopting a trapezoidal shape for the siphoning region instead of a rectangular one to improve consistency of the microfluidic chip fabrication (see Section “Fabrication of Microfluidic Chip”). Additionally, the design is symmetric on both sides to ensure uniform droplet pressure distribution. Control of the siphoning region is achieved via a solenoid valve connected to the siphoning region outlet tubing, functioning as an on–off switch (Figure S14, Supporting Information). When the solenoid valve is closed, the siphoning region is inactive, and droplets flow through the main channel at their initial velocity and spacing (**Figure** **4**a). When the valve is opened, the siphoning region becomes active, slowing the droplets, allowing them to come into contact and form bilayers using the droplet interface bilayer (DIB) method (Figure 4b, Movie S2, Supporting Information). A short meander channel (19 mm in length) follows the siphoning region to stabilize the bilayer adhesion before the droplets exit the microfluidic chip.

Once the droplets exit the chip, they travel through the main channel outlet tubing connected to a three‐axis stage for deposition (Figure S15, Supporting Information). Our experiments demonstrated that the droplet thread assumes various packing configurations, depending on the tubing's inner diameter ratio to the droplet's diameter and the input pressure of the continuous oil phase. These findings align with previous research on droplet packing in microfluidic systems.^[^ ^43^ ^]^ For example, when the ratio of the tubing's inner diameter to the droplet's diameter is approximately one, the droplets exit as a single thread (Figure 4c, Movie S3, Supporting Information). This single droplet thread can also be obtained by increasing the pressure of the continuous phase when the tubing's inner diameter exceeds the droplet diameter. However, a higher pressure, and hence a higher flow rate (Equation 1), may cause the droplet thread to split into smaller segments as it moves through the tubing. Therefore, to ensure the formation of a single thread, it is advisable to choose an inner tubing diameter that closely matches the droplet diameter. If the ratio of the tubing's inner diameter to the droplet's diameter exceeds one, various packing configurations may occur based on this ratio.^[^ ^43^ ^]^ In our experiments, the tubing's inner diameter is 0.8 mm and the droplet average diameter is 0.465 mm. For an oil input pressure of 65 mbars, the ratio between the tubing's inner diameter and droplet diameter is 1.72, resulting in an alternating zig–zag configuration (Figure 4d, Movie S4, Supporting Information).^[^ ^43^ ^]^ To demonstrate the stability and endurance of the bilayer during printing, we used the zigzag packing configuration in subsequent experiments. Additionally, we determine the droplet output accuracies (DOA), defined as the percentage of correct droplet sequences, between the droplet sequence generated internally in the microfluidic chip and the sequence exiting the nozzle (DOA_CN_) as well as the accuracy of the sequence depositing from the nozzle compared to the sequence in the mold (DOA_NM_). In this work, the DOA_CN_ is 81.1 ± 17.0 % and the DOA_NM_ is 97.5 ± 4.3 %, conveying that most of our errors occur as the droplet exit the chip (more information provided in Supporting Information Section S7: Equation S4 and Figures S9 and S10). We attribute most of these errors in droplet output accuracy to droplet fusion and sequence rearrangements that occur from pressure differences during the off‐ and on‐transition states of the siphoning region (Section S7, Supporting Information).

**Figure 5:**
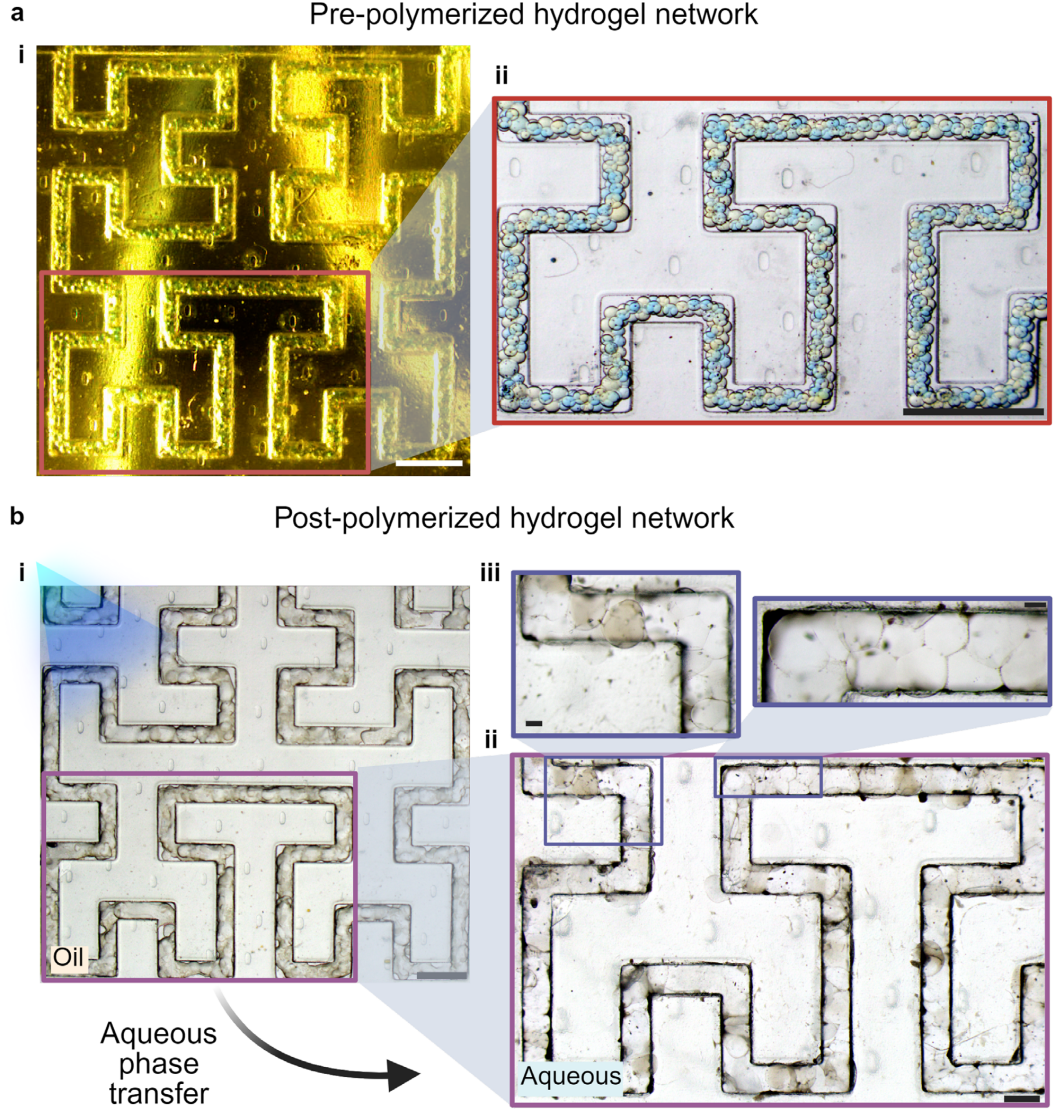
a) A completed 3rd‐order Hilbert Curve after printing, displaying droplet interface bilayers before curing. i) The image is taken immediately post‐print. The image has a yellow tint as the lighting conditions during printing are dim to prevent photopolymerization of the hydrogels (Scale bar = 3 mm). ii) The printed droplet network under a stereoscope before polymerization, displaying the droplet interface bilayers (Scale bar = 2 mm). b) Polymerization and phase transfer of droplet network. i) The hydrogels are polymerized using ultraviolet light in oil (Scale bar = 2 mm). ii) The cured hydrogel 3rd order Hilbert curve is placed in an aqueous solution (Scale bar = 1 mm). iii) The complete structure retains the shapes provided by the DIBs (Scale bars = 200 µm). The image contrast was adjusted for enhanced visualization without altering the original data.

After traveling through the main channel outlet tubing, the droplet thread is deposited into an oil vat. Deposition geometry and speed are controlled by a custom‐made nozzle attached to a 3‐axis stage (Figure S15; factors impacting printing resolution are discussed inSection S7, Supporting Information). The nozzle deposits the droplet thread at a constant velocity of 2.3 mm s^−1^. As a demonstration, we printed a 3rd‐order Hilbert curve (**Figure** **5**, Movies S5 and S6, Supporting Information), a space‐filling fractal commonly used in computational design.^[^ ^44^ ^]^ In this context, it showcases the system's ability to deposit droplets continuously with high positional accuracy, even during directional changes (demonstration of multi‐layered 3D printing is shown in Figure S13, Supporting Information). During printing, the droplets remain in their pre‐polymerized hydrogel phase (Figure 5a–i). After printing, ultraviolet (UV) light is used to cure the droplets at a distance of 7.5 cm for 60 min, which disrupts the lipid bilayers and fuses the droplets into a continuous multi‐material hydrogel structure (Figure 5b–i). This disruption is evident in dye dilution: prior to curing, the lipid bilayers prevent dye diffusion (Figure 5a–ii); however, after UV exposure, the dye freely diffuses through the now‐continuous hydrogel network (Figure 5b–i). The printed structure can then be transferred into aqueous environments (Figure 5b–ii), and the geometry defined by the original DIBs remains intact, confirming the creation of a continuous multi‐material hydrogel (Figure 5b–iii). The stiffness of our continuous hydrogel structure, which has a Young's modulus range of 0.2–1.82 kPa matching the stiffness range of similar hydrogel compositions, demonstrates that the interfacial adhesion between cured droplets maintains robustness of the final continuous hydrogel structure (Section S8, Figures S11 and S12, Supporting Information).^[^ ^45^, ^46^, ^47^, ^48^ ^]^

## 3 Conclusion

This study presents a novel microfluidic approach for generating multi‐material hydrogel threads suitable for 3D printing diverse structures via extrusion methods. We introduce a pioneering, continuous microfluidic oil siphoning technique, which facilitates the interaction of droplets of varying compositions and allows them to form a continuous thread post‐generation within the microfluidic chip. To prevent droplet merging, we infused the oil phase with phospholipids, creating a lipid monolayer on the surface of each droplet that transitions to a lipid bilayer upon contact with another droplet. By manipulating various design parameters, we demonstrated the ability to control both the oil extraction flow rate and the structural configurations of the multi‐material threads, enabling the study of dynamical droplet behavior in a wide range of applications, including assays, coalescence studies, and droplet capture. Moreover, the continuous oil siphoning microfluidic technology significantly enhances multi‐material 3D printing capabilities by facilitating the swift voxelated extrusion of low‐viscosity and aqueous inks—a feat not yet fully realized in the field, while also augmenting resolution and complexity for hydrogel applications through repeatable gradient structures.^[^ ^6^, ^49^, ^50^ ^]^ An alternating sequence of voxelated droplets in the thread promotes the formation of structures with varying stiffness and stimuli‐responsiveness to expand the movement, robustness, and control of soft actuators.^[^ ^51^, ^52^ ^]^ The voxelated thread provides additional sensing sites for improved resolution and versatility for hydrogel sensors, while changes in the packing configuration of the thread during printing can incorporate additional encryption levels in pH‐responsive data encrypted hydrogels.^[^ ^52^, ^53^ ^]^ Similarly, repeatable alternating sequences of the hydrogel thread enables the scalable fabrication of complex DIB‐assembled hydrogel soft power sources to be utilized in neuronal network activity and tissue stimulation.^[^ ^17^, ^18^ ^]^ Overall, this method enables scalable printing, akin to traditional extrusion techniques, ensures high cell viability comparable to inkjet printing, and enables the fabrication of repeatable gradient structures highly desired in soft robotic actuation, sensing, and medical applications.^[^ ^50^ ^]^

Looking ahead, several refinements could further enhance this technology. One key improvement would involve integrating microfluidic valves into the chip design. This addition would facilitate the scalability of material diversity, provide better control over the sequence of material deposition, and allow for adjustments in droplet size based on varying fluid properties.^[^ ^54^, ^55^, ^56^ ^]^ Another area for optimization lies in selecting continuous motors for the 3‐axis stage. The current reliance on linear discrete motors presents challenges in synchronizing movements for nonlinear geometries, given their independent control and time delays in coordinated transitions. By transitioning to continuous motors, we could enable the fabrication of more complex, nonlinear structures, enhancing the capabilities of this innovative printing method. Additionally, shifting from the reliance on 3D‐printed molds as structural supports to alternatives such as dissolvable support baths, will ease fabrication and post‐print processing of final 3D geometry.^[^ ^57^, ^58^ ^]^ Currently, this fabrication technique requires 3D resin molds to maintain the complex structure of the pre‐polymerized hydrogel network, unlike other droplet printers which are capable of producing mold‐free pre‐polymerized 3D structures.^[^ ^16^ ^]^ Without the molds, the thread will not retain its position, as the oil flow from the nozzle movement will cause migration of the thread due to the low viscosity of the pre‐polymerized hydrogels (Section S9, Supporting Information). Furthermore, it is difficult to remove the complete polymerized 3D structure from the resin mold without damaging the final print. To improve structural support during printing and ease post‐processing, partial polymerization of the hydrogel thread before deposition and shifting to dissolvable support baths will elevate this technology.^[^ ^57^, ^59^, ^60^ ^]^ In summary, this research lays the foundation for advancing microfluidic techniques and multi‐material 3D printing, paving the way for future innovations in the fabrication of high‐performance structures and devices.

## 4 Experimental Section

### Fabrication of Microfluidic Chip

Microfluidic chips were designed in Autodesk Inventor (Figure S1, Supporting Information) and 3D printed with an Anycubic M3+ DLP printer. The resin mold was washed in an IPA bath for 10 min and then air‐dried. The resin mold was then cured in a UV oven at 60 °C for 60 min (LED Lamp 405 nm, 125 W) followed by a 16 hour cure in an oven at 70 °C.

The 3D printed resin mold was then cast with 10:1 PDMS (Dow Slygard 184), vacuumed to remove the air bubbles, and placed in an oven at 70 °C for 2 h. Additionally, a thin layer of PDMS was spin‐coated (500 rpm for 20 s, followed by 6000 rpm for 50 s) on a glass slide. The glass slide was also placed in the oven for 2 h at 70 °C. After 2 h, the PDMS microfluidic chip and glass slide were treated with a plasma cleaner (Harrick Plasma PDC‐001) at high RF levels for 60 s and bonded together. Finally, the entire assembly was placed in the oven for another 2 h at 70 °C.

For improved consistency and accuracy of the microfluidic chip, the rectangular siphoning region was altered to a trapezoidal siphoning region. When 3D printing the molds with rectangular siphoning regions, specifically for wide sections of the siphoning region, the slits would either be deformed or absent due to bleeding from the UV light of the DLP printer. Additionally, when washing the slits with IPA, not all the resin was removable, creating inaccurate and inconsistent slit pathways. With a trapezoidal siphoning design, these challenges are greatly reduced.

### Preparation of Hydrogel Solutions

All chemicals were purchased from Sigma–Aldrich. Three different hydrogel compositions were formulated: Composition 1 consisted of 2.86M of Acrylamide, 1.6 mM N,N'‐Methylenebis(acrylamdie) further forth known as “bis”, 34 mM 2‐hydroxy‐4'‐(2‐hydroxyethoxy)‐2‐methylpropiophenone (Igracure‐2959), and 0.05 mM Xylene Cyanol FF blue dye. Composition 2 consisted of 2.85 M of Acrylamide, 1.6 mM bis, 2.0 mM Poly(ethylene glycol) diacrylate (PEGDA, average MW 700), 33.9 mM Igracure‐2959, and 0.05 mM orange G biological dye. Composition 3 consisted of 2.56 M of Acrylamide, 1.5 mM bis, 30.4 mM Igracure‐2959, a one‐to‐one mixture of 0.11 mM of xylene cyanol ff blue dye and 0.11 mM orange G biological dye mixed together to form a green dye. The solvent for composition 3 consisted of 5 wt% of poly(vinyl alcohol) (PVA, average MW 30 000–70 000) stock solution instead of only Milli‐Q water as used for compositions 1 and 2.

### Preparation of Lipid–Oil Solution

25 mg mL^−1^ of 1,2‐diphytanoyl‐sn‐glycero‐3‐phosphocholine (DPhPC) dissolved in chloroform were purchased from Avanti Polar Lipids. The lipids were aliquoted into vials containing 25 mg DPhPC. To prepare lipid–oil solution, the aliquoted vial was placed under a light stream of compressed dry air (CDA) until all the chloroform has evaporated. Then, the vial was placed under vacuum (vacuubrand ME 1C) at a pressure of ‐14.5 psi for 60 min. 6.25 mL of undecane oil (purchased on Sigma–Aldrich) was added to the vial and slightly mixed to create 4 mg mL^−1^ DPhPC‐Undecane lipid oil stock solution. This was stored in the fridge (2–4 °C) until ready for use. For experimentation, a 1:1 mixture of the 4 mg mL^−1^ DPhPC‐undecane stock solution was mixed with silicone oil AR20 (purchased on Sigma–Aldrich) to create a final solution of 2 mg mL^−1^ DPhPC in 1:1 undecane–silicone oil solution.

### Control of Solenoid Valve

A solenoid valve was used to enable and disable the siphoning region in the microfluidic chip. The solenoid valve (PreciGenome, 2‐way normally closed, 1/32'' ID x 3/32'' OD, 12V) was attached to the outlet tubing of the siphoning region (Figure S15, Supporting Information). The valve was controlled through an Arduino Uno programmed through a custom made MATLAB code (Figure S14, Supporting Information).

### Programming and Printing of Droplet Threads

The microfluidic chip was controlled through a pressure‐driven flow. A pressure controller (OB1 MK3+) was purchased from Elveflow Microfluidics with 4 pressure channels. For this study, all the pressure channels used have the same input pressure range of 0–2000 mbars. The oil reservoir was controlled by one channel, two reservoirs with compositions 1 and 2 (Section “Preparation of Hydrogel Solutions”) were controlled by another channel, and composition 3 (Section “Preparation of Hydrogel Solutions”) was controlled by a third channel due to the gradual increase in viscosity. For printing, the oil reservoir had an input pressure of 65 mbars, compositions 1 and 2 had an input pressure of 30 mbars, and composition 3 had a input pressure of 35 mbars. All reservoirs were connected to the chip with a combination of PTFE tubing (Elveflow Microfluidics 1/16'' OD and 1/32'' ID) and resistance tubing (Elveflow Microfluidics, ID 175 µm). The length of the tubing for all reservoirs was 88 cm of PTFE tubing, 5.2 cm of resistance tubing, and 33 cm of PTFE tubing. To control the deposition of the multi‐material hydrogel thread, a custom 3D printed nozzle fixture was attached to a Thorslab 3‐axis stage MTS50‐Z8 controlled by KDC101 brushed DC motor (Figure S15, Supporting Information). The 3‐axis stage was programmed by a custom made LabView program.

### Curation of Printed Hydrogel Structure

To cure the printed pre‐polymerized hydrogel structure, the oil vat was left to rest for 10 min at room temperature to provide the lipid bilayers time to stabilize post‐deposition. The oil vat solution was then moved into a custom‐built nitrogen chamber and was purged with nitrogen for 45 min. Afterwards, the vat was placed under a UV light (Mineralight, Analytik Jena, with 302 nm wavelength and 50 W of power) at a distance of 7.5 cm for 60 min. After the hydrogels polymerized, the 3D printed resin mold on which the hydrogels were printed on, is carefully removed from the oil vat. The residual oil is washed multiple times by gently pipetting DI water over the hydrogels. Finally, the entire hydrogel structure is moved into an aqueous container.

### Determination of Extracted Oil Flow Rate for Varying Siphon Channel Dimensions

A custom‐made MATLAB code was written to evaluate the change in the oil siphon flow rate as the geometrical dimensions of the siphoning region changed based on the circuit diagram in Figure S4, (Supporting Information). To experimentally validate the code, a control rectangular siphoning channel geometry, based on preliminary data, was chosen with the following dimensions: 3 mm in length, 0.3 mm in height, and 0.2 mm in width. When modifying one of the dimensions of the siphoning channel, the other dimensions remained constant with the dimensions of the control geometry. For all the chips tested, the oil input pressure was 50 mbars and the aqueous input pressure was 30 mbars. The solenoid valve controlled the siphoning region and cycled between off and on stages, each lasting 90 s. Each chip was cycled for 3 cycles. Videos were captured during experimentation with Thorslab video cameras (CS895CU and CS165CU). To determine the extracted oil flow rate, a custom made python code with OpenCV library was used for image processing. The difference in flow rate was calculated between the off and on state for each cycle and averaged for the three cycles.

### Mechanical Characterization of Polymerized Hydrogel Thread

Uniaxial tension measurements of the polymerized hydrogel thread were measured with MTS Criterion Model 43 with a 50 N load cell (MTS Load Cell LSB.501 D) (Section S8, Supporting Information). The samples were pulled at a rate of 2 mm s^−1^ until failure.

## Conflict of Interest

The authors declare no conflict of interest.

## Supporting information

Supporting Information

Supplemental Movie 1

Supplemental Movie 2

Supplemental Movie 3

Supplemental Movie 4

Supplemental Movie 5

Supplemental Movie 6

## Data Availability

The data that support the findings of this study are available from the corresponding author upon reasonable request.
