# Supplementary material for: Multi‐Material Droplet‐Based Hydrogel Threads for Extrusion 3D Printing
Source: Small Methods. 2025 Nov 2;10(5):e00928. doi: 10.1002/smtd.202500928 (PMC12972237; doi:10.1002/smtd.202500928)
Supplement: Supplementary file 1 — Supporting Information [file SMTD-10-e00928-s006.pdf]

Supporting Information

Multi-Material Droplet-Based Hydrogel Threads for Extrusion 3D Printing

*Dor Tillinger, Nicholas X. Armendarez, Joseph S. Najem**

Movies

Movie S1. Alternating T-junction droplet generation of three different droplet types.

Movie S2. Formation of the droplet thread through dynamic oil siphoning. When the siphoning region is off, the droplets maintain their spacing based on their generation rate. When the siphoning region is activated, the oil is siphoned out of the main channel, bringing the droplets into closer proximity and allowing them to touch. When the droplets come into contact, they form lipid bilayers between them and assemble into a droplet thread.

Movie S3. Deposition of a single droplet thread consisting of two ink types.

Movie S4. Deposition of droplet thread in a zig-zag configuration consisting of two ink types.

Movie S5. Side view of the multi-material droplet thread being deposited. The lipid bilayers maintain their assembly as the thread is deposited through various processes turns.

Movie S6. Bottom view of multi-material printing of the droplet thread.

S1. CAD design of the final microfluidic chip

The final design of the microfluidic chip and its dimensions are presented in Figure S1. Inset A illustrates the dimensions of the aqueous inlets, all of which are identical. Inset B depicts the measurements related to the siphoning region, while inset C reveals the dimensions of the slit pathways. In Figure 3, adjustments to the various siphoning region design dimensions highlighted that the siphoning channels possessed a rectangular shape, contrasting with the trapezoidal design shown in the final CAD rendering. The trapezoidal shape was selected to enhance fabrication consistency (experimental subsection 4.1). Furthermore, experiments indicated that the trapezoidal design resulted in less droplet deformation and coalescence compared to the rectangular siphoning channel geometry.

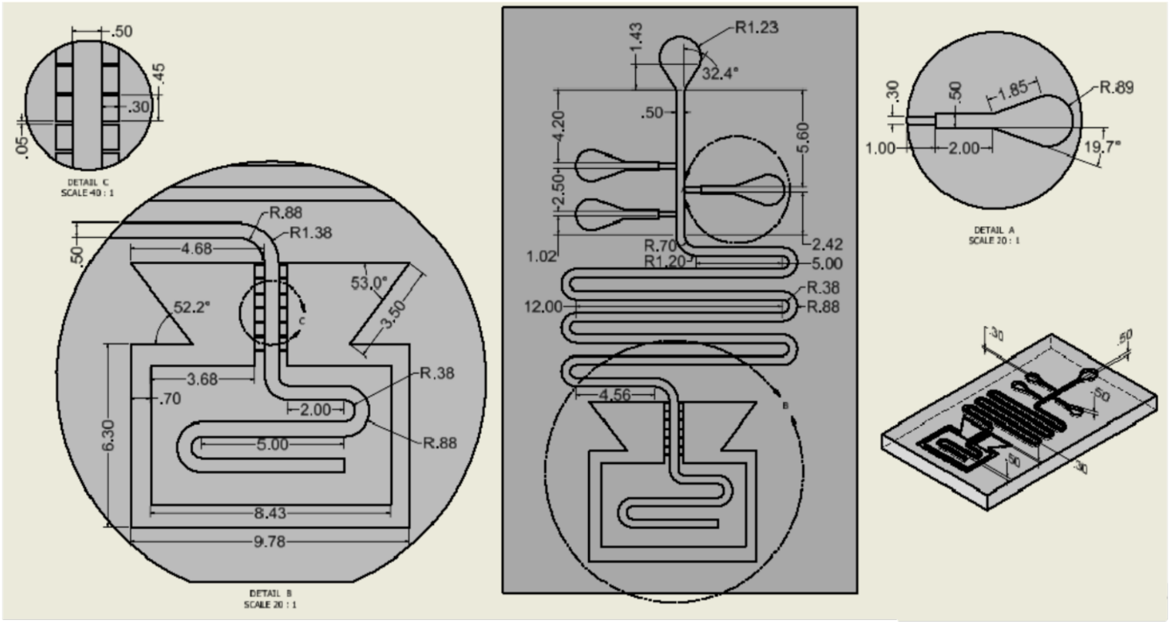

Figure S1: 3D CAD drawing of the finalized microfluidic chip design. All dimensions are in mm.

S2. Consistent droplet generation and droplet spacing

Microfluidic chips are utilized for droplet generation due to the consistency and monodispersity of droplet sizes. The sizes of droplets can be controlled based on channel dimensions and input pressures. For the finalized chip dimensions illustrated in Figure S1, droplet volumes were measured for a single droplet type in the meander region. Droplet volumes were evaluated over four cycles of 90-second intervals during which the siphoning region was inactive (no oil was extracted) and active (oil was extracted). The average droplet volume was found to be 52.6 ± 4.9 nL (an average droplet diameter of 465 ± 15 µm). Additionally, we quantified the center-to-center distance between droplets as they travel through the meander region when both

siphoning was inactive and active. The average droplet spacing was determined to be 1.95 ± 0.08 mm.

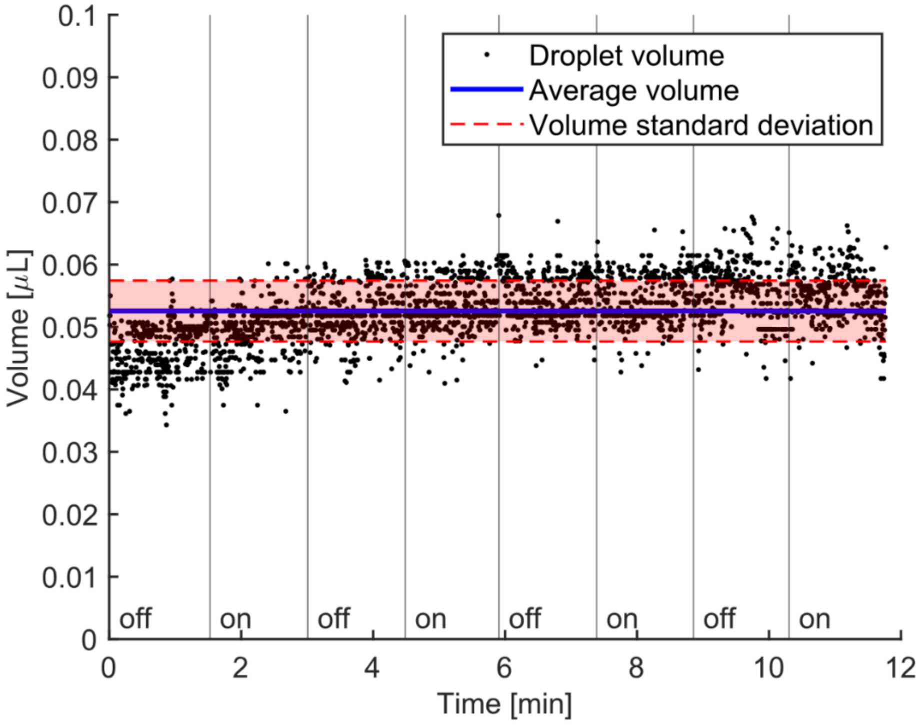

Figure S2: Droplet volume over time. The droplet volume post-generation but before the siphoning region is consistent regardless of whether the siphoning region is inactive (off) or active (on). The transient region at the start of the droplet generation is constant after 120 seconds.

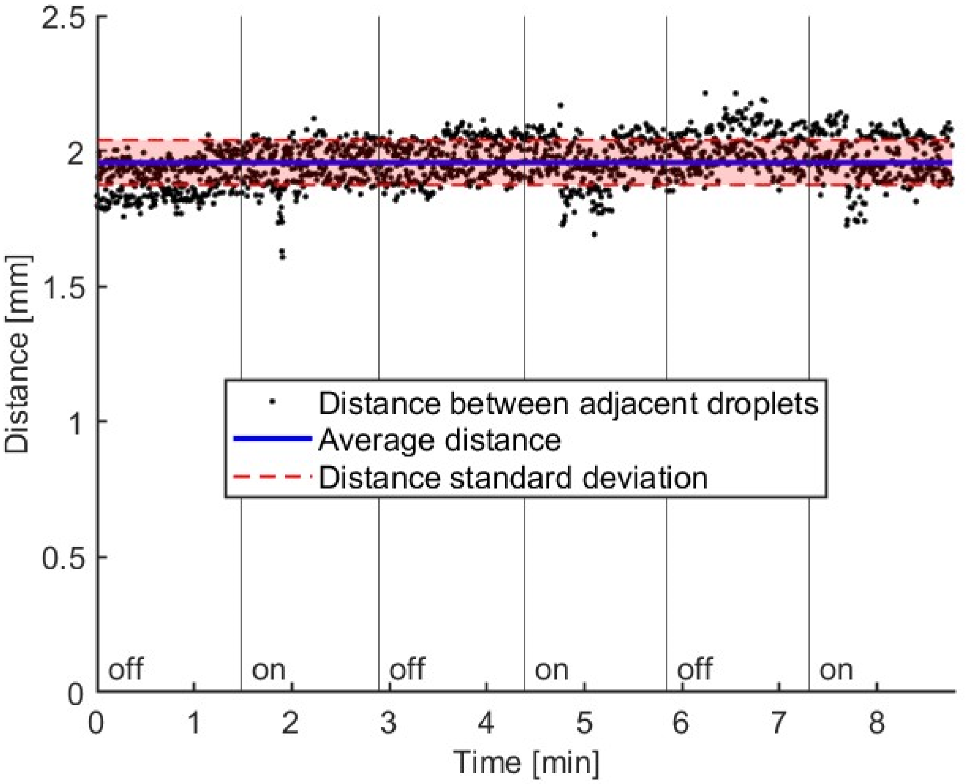

Figure S3: Center-to-center distance between droplets in the meander region. Before droplets enter the siphoning region, The spacing between droplets remains consistent regardless of whether the siphoning region is inactive (off) or active (on).

S3. Analogous circuit diagram of microfluidic chip

In microfluidics, viscous forces dominate inertial forces due to the miniature characteristic length scale, typically on the micron scale, and the low fluid velocities [1, 2, 3]. As a result, fluid flow in microfluidics is almost always laminar due to the low Reynolds number [1, 2, 3]. Furthermore, the flow in a microfluidic chip is primarily influenced by the pressure gradient from the upstream to the downstream section, the fluid flow rate, and the hydraulic resistance determined by the geometric features of the chip [1, 2, 3], as described in the following relationship:

$$\Delta P_{hyd} = QR_{hyd} \quad \text{(S1)}$$

where ΔP_{hyd} is the hydraulic pressure difference, Q is the fluid flow rate, and R_{hyd} is the hydraulic resistance [1, 2]. For a rectangular microfluidic geometry, in which the width is much greater than the height, the hydraulic resistance is commonly defined as:

$$R_{hyd} = \frac{12\mu L}{h^3 w}\frac{1}{1-0.63\frac{h}{w}} \quad \text{(S2)}$$

where μ is the fluid viscosity, L is the length of the channel, h is the height of the channel, and w is the width of the channel [1, 2].

Due to the laminar regime, the relationship among the variables in Equation S1 is analogous to the ohmic relationship found in electrical circuits [1, 2, 3]. Therefore, the pathway designs in microfluidic chips can be arranged in series and parallel configurations, allowing for the control of fluid flow rates in specific sections by adjusting the local hydraulic resistance through geometric designs [1, 2, 3].

In Figure S3, the finalized microfluidic chip is represented as an analogous circuit diagram. This diagram helps visualize the regions in the system that have the largest changes in flow rate. Each pressure point, highlighted in orange, represents a region in the chip where a significant pressure change occurs. Highlighted in a dashed red square are the resistances associated with the siphoning region. The bottom left-hand-side inset overlays the resistances associated with each specific component in the siphoning region. When changes are made to the geometric features of a component, the resistance of the chip at the siphoning section will adjust accordingly. Thus, that region is symbolized as a variable resistance. Equations S1 and S2 are used to calculate the changes in hydraulic resistance and flow rates based on the chosen geometrical dimensions. When designing the geometry of the siphoning region, it is essential to consider that during siphoning operations, droplets will aggregate in the main channel as they adhere to form a thread. The pressure will build up in the main channel between the outlet pressure and P_2 (as shown in Figure S3), thereby increasing the resistance in the main channel. To ensure the successful formation of droplet threads without droplet deformation or coalescence, which is undesirable, it is important to design the dimensions of the siphoning region while incorporating the additional resistance formed from the droplet build-up.

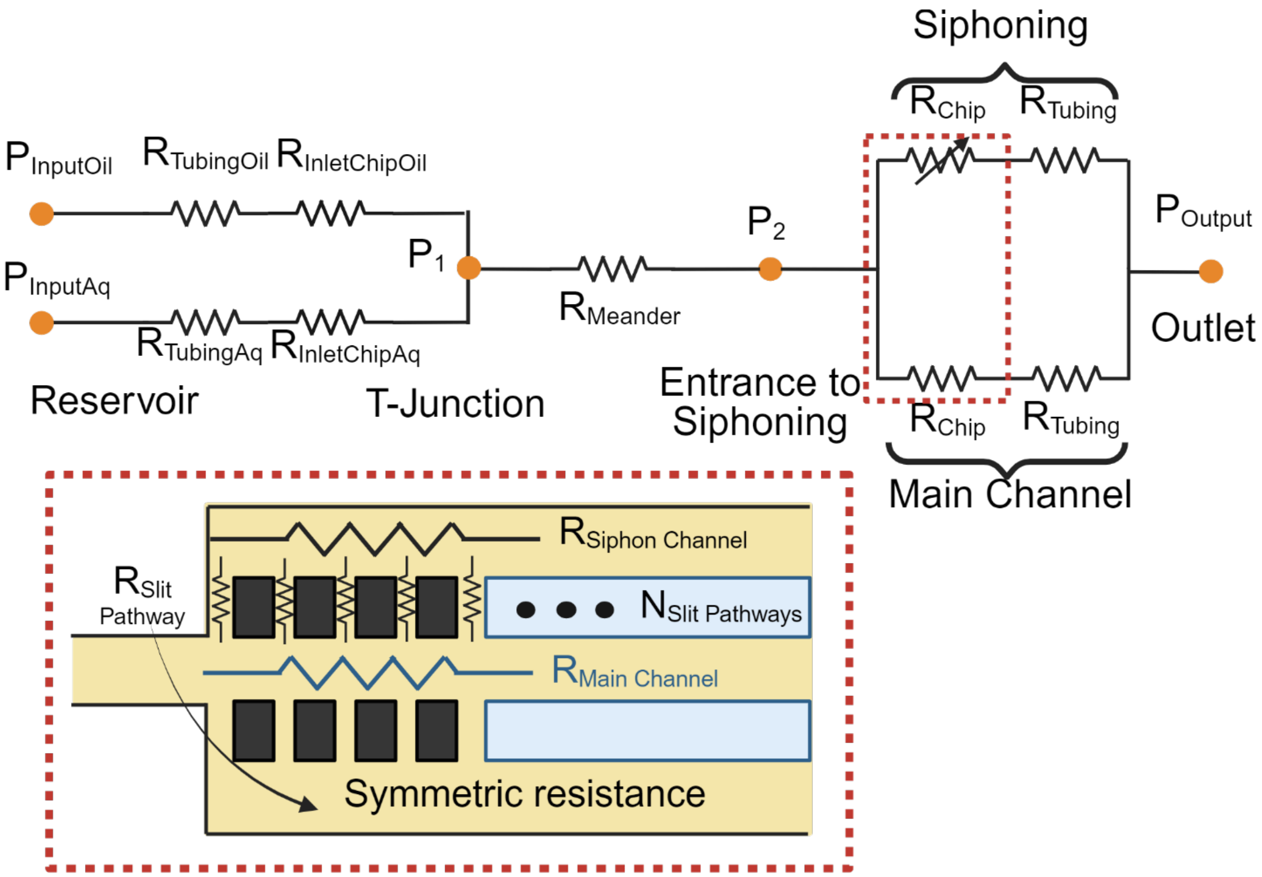

Figure S4: Analogous microfluidic circuit diagram. The orange dots represent all the major pressure droplets occurring along relevant sections of the microfluidic chip. Highlighted in the red dash square is the resistances labeled along its corresponding microfluidic feature.

S4. Applying the generalized hydraulic resistance equation when varying the geometrical dimensions of the siphoning channel

To evaluate the change in the oil siphon flow rate as the geometrical dimensions of the siphoning region are changed, a custom MATLAB code was written. The flow rate can be calculated by dividing a localized pressure gradient of a specific region in the microfluidic chip by its hydraulic resistance. Equation S2, in section S3, describes the typical hydraulic resistance equation of a rectangular geometry in a microfluidic chip. However, this equation assumes that the width of the channel is sufficiently larger than the height [1]. When analyzing the impact of increasing one of the geometrical features of the siphoning channels, there is a region in which the width and height dimensions are of similar magnitudes. For this region, Equation S2 does not accurately predict the effect of oil siphoning, as the assumption for Equation S2 is nullified [1].

To precisely determine the resistance of the siphoning channels as the geometrical dimensions of the region are modified, a more generalized resistance equation is derived from calculating

the flow rate of a Poiseuille flow in a rectangular channel [1]. The generalized resistance equation, Equation S3, contains a Fourier expansion term which more accurately estimates the resistance when the width and height dimensions are close in value [1].

$$R_{rect\ gen} = \frac{12\mu L}{h^3 w} \frac{1}{[1 - \sum_{n_{odd}}^{\infty} \frac{1}{n^5} \frac{192}{\pi^5} \frac{h}{w} \tanh\left(n\pi \frac{w}{2h}\right)]} \quad (S3)$$

For calculating the effect of the oil siphoned by one of the geometrical dimensions (either width or height) of the siphoning channels, the other two geometrical features are kept constant. Two boundary conditions are applied when calculating the resistance for the width or height dimensions. One of the conditions is when the varying dimension is smaller than the fixed dimension and the other is when the varying dimension is equal to or greater than the fixed dimension. For example, if you want to determine the change in resistance of the siphoning channel based on varying the width from 0.01 mm to 2 mm. The length and height are fixed at specific values, in this case 3.5 mm and 0.3 mm respectively. The generalized resistance equation will be applied for the two boundary conditions. The resistance is calculated for each dimension when the width is less than the fixed height dimension (0.3 mm), $0.01 \leq w < 0.3$ mm, and calculated again when the width is equal to or greater than the height dimension, $0.3 \leq w < 2$ mm. After the resistance is calculated, the extracted oil flow rate is calculated using Equation S1 with the chosen input pressures. Figure S3 expands upon Figure 3c, by adding insets to illustrate the steep growth in siphoned oil flow rate when the width dimension is smaller than the height dimension, and the shallower growth in flow rate that is observed when the width is greater than the height dimension. This calculation is repeated when varying the height dimension, while keeping the width and length dimensions constant (Figure S5). The generalized resistance equation is also used to calculate the resistance for varying length of the siphoning channel when the width and height dimensions are fixed but similar in magnitude.

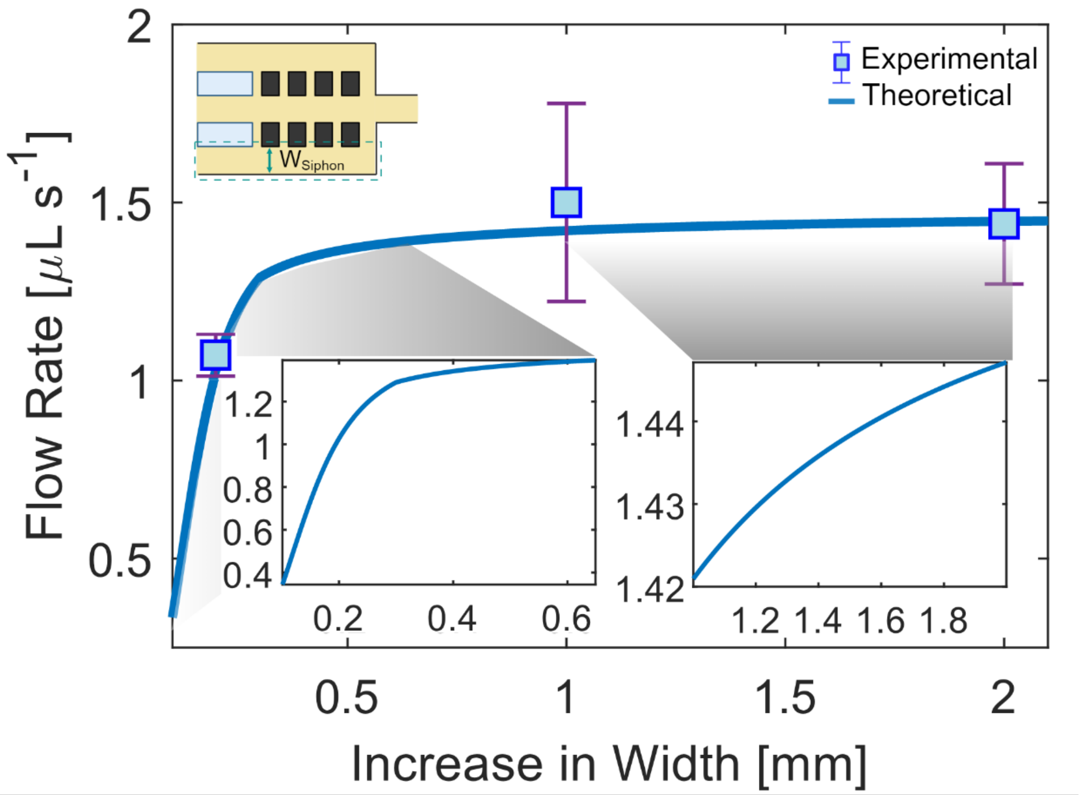

Figure S5: Oil siphoned flow rate as the width of siphoning channel increases. When the width is less than the height (0.3 mm), there is a steep growth (bottom left inset). When the width is greater than height dimension (0.3 mm), the flow rate increases at shallower rate toward an asymptotic value (bottom right inset).

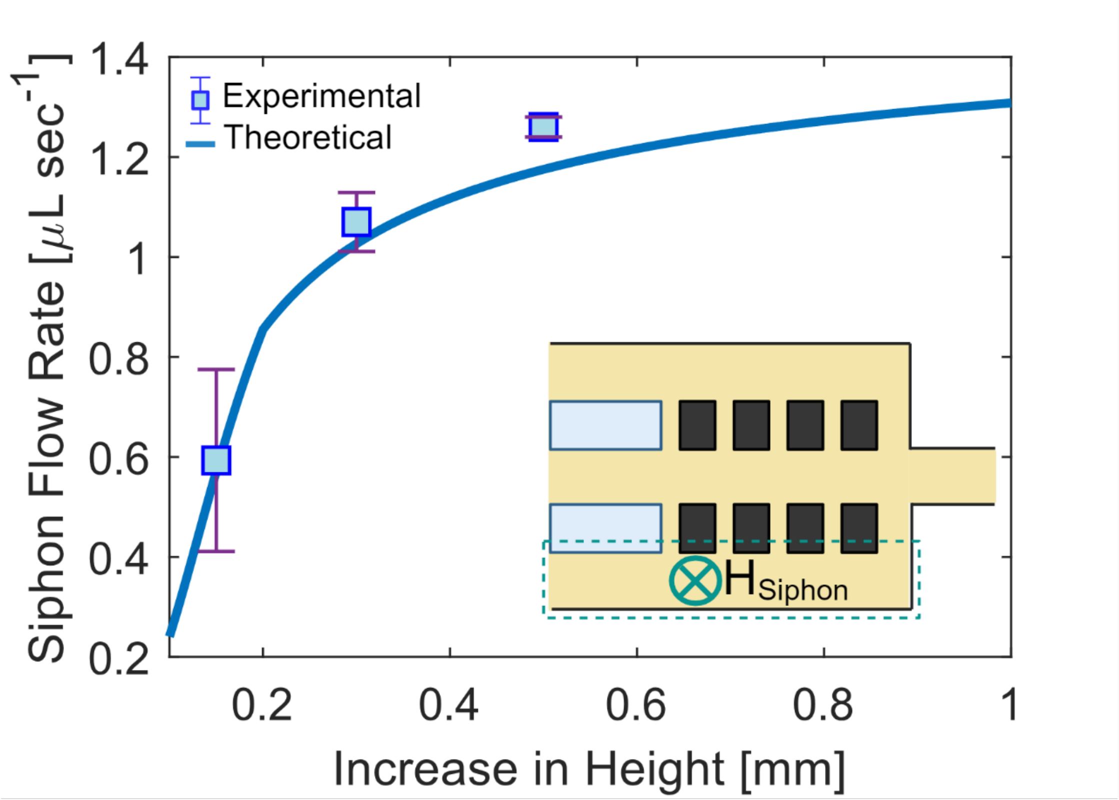

Figure S6: Oil siphoned flow rate as height of siphoning channel increases. The same trend is observed here as seen with the width. When the height is less than the width dimension (0.2 mm), there is a steep growth. When the height is greater than the width dimension (0.2mm), the is a shallower asymptotic growth.

S5. Impact of additional slits on oil flow rate

When increasing the length of the siphoning region, additional slit pathways are created as more pillars are added to compensate for the augmented length of the siphoning channel. Based on the circuit diagram (Figure S2 inset), additional slit pathways are added in parallel, resulting in a minimal increase in the net resistance of the siphoning region and thus a minimal impact on the siphoned oil flow rate. To demonstrate this, we calculated the oil flow rate as the number of slit pathways increase for a fixed siphoning channel length of 9 mm. It is observed that the number of slit pathways has a negligible impact on the oil siphoning flow rate (Figure S6).

Our slit pathway dimensions are 0.3 mm in length, 0.3 mm in height, and 0.05 mm in width (Figure S1). When calculating the oil siphon flow rate as the number of slit pathways increased, we used the general resistance equation. We did this to achieve more precise results and to maintain consistency when simulating trends in extracted oil flow rates.

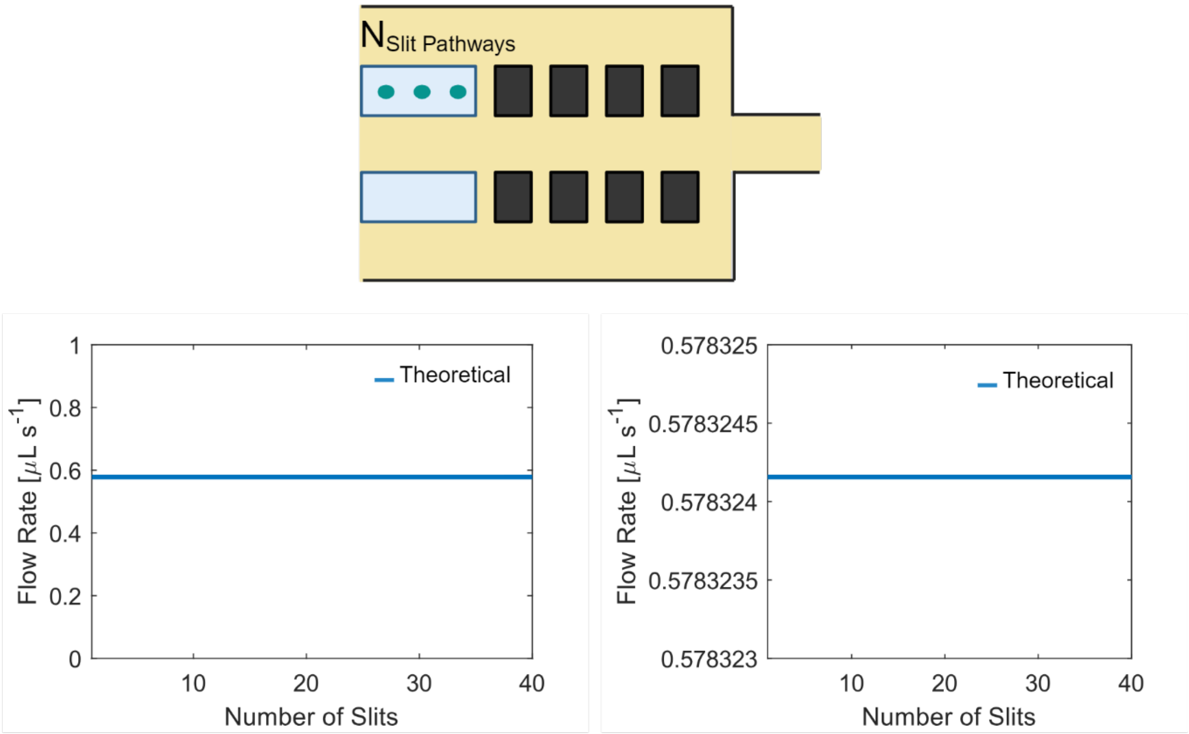

Figure S7: Simulated results for the oil siphoning flow rate as the number of slit pathways in the microfluidic chip increase for a fixed length of 9 mm. There is a negligible effect on the amount of oil extracted as the number of slits increases. The right graph magnifies the scale bar of the y-axis to demonstrate the negligible change in flow rate.

S6. Device-to-device variation of oil siphoning

For Figure 3, the statistics for the experimental validation point are determined by measuring the flow rate between 3 different off-on cycles in the siphoning region for a single chip. To demonstrate that the extracted oil flow rate is repeatable across different devices, measurements of the extracted oil flow rate were taken for three off-on siphoning cycles using three different chips with the same siphoning dimensions. In Figure S5, the mean and standard deviation of the extracted oil flow rate for three different off-on cycles are represented for three microfluidic chips with a rectangular width dimension of 2 mm.

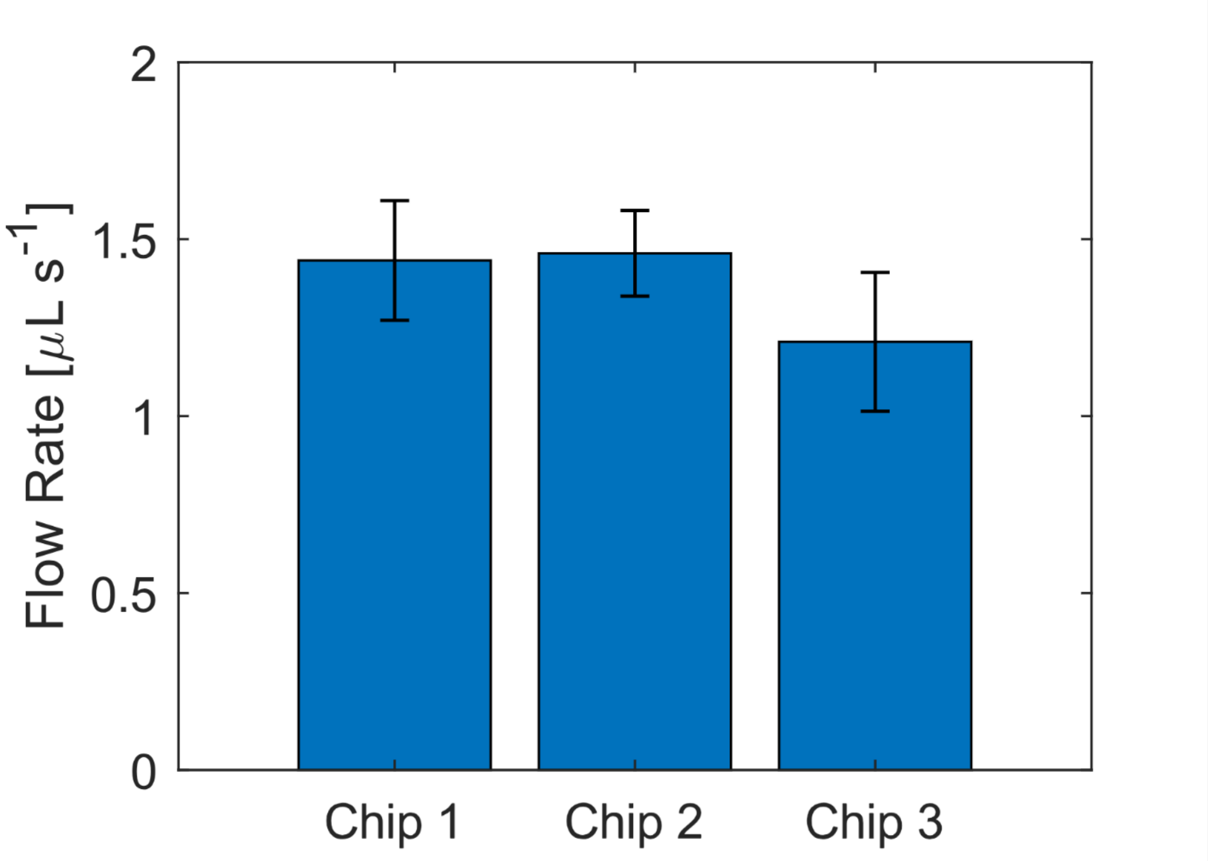

Figure S8: Device-to-device variation of the oil flow rate being siphoned between 3 microfluidic chips with a siphon channel width of 2 mm. The mean and standard deviation represent the amount of oil being siphoned between each off-on cycle of the siphoning region for three cycles.

S7. Factors impacting printer resolution

There are a few key design metrics and factors that heavily impact the resolution and capabilities of the multi-material hydrogel thread and final print. The metrics are summarized in Table S1 and are described in more detail as follows:

Minimum Droplet Diameter (MDD): The minimum droplet diameter (MDD) determines the minimum feature size and scalability of the printed structure. For forming microemulsions of stable aqueous droplets with surfactants in oil, which is required for the formation of the multi-material thread, the minimum droplet diameter is greater than 100 nm [30-32]. When the droplet diameter falls below 100 nm, additional forces such as line tension become non-negligible, which can cause droplet instabilities and fusion [8].

Practically, there are additional limitations to the MDD steming from the fabrication of the microfluidic chip. The droplet diameter should be similar in size to the channel width of the microfluidic chip so that the siphoning region only extracts oil without letting droplets deform and enter the siphoning channel. The smallest geometric feature size of the microfluidic chip used in this work is determined by the XY pixel resolution of the Anycubic DLP printer, which prints the microfluidic molds (Experimental section 4.1). This printer's XY pixel resolution is 34.4 μm, constraining the width of the slit pathways

in the siphoning region. For consistency across fabrications, we fabricated the slit pathways at 64.8 µm (2 pixels) in width. However, due to resin bleeding during curing, the final slit pathway width measures approximately 50 µm (Figure S1). Because of the slit pathway size, we designed the main channel to be 500 µm wide, ensuring that the Laplace pressure needed to deform a droplet into the siphoning pathway exceeds the pressure droplets experience as they pass through the siphoning region.

To ensure the droplet size is similar to the width of the main channel in the microfluidic chip, the ratio between the oil (continuous phase) and aqueous (disperse phase) input pressures was adjusted to produce droplets of comparable size (Experimental section 4.5). Increasing the ratio of the continuous to disperse phase input pressures results in smaller droplets than the width of the main channel and increases the likelihood of deformation into the siphoning pathways [23-24]. In this work, the minimum droplet diameter is 465 ± 15 µm (Figure S2).

Droplet Output Accuracy (DOA): We define two droplet output accuracy (DOA) metrics, one between the droplet sequence generated in the microfluidic chip and the output of the nozzle (DOA_{CN}) and one between the droplet sequence at the output of the nozzle compared to the sequence of droplets deposited in the mold (DOA_{NM}). A single DOA metric between the chip to the mold is difficult to analyze as our camera set up is fixed (Figure S15), which prevents examining the sequence in the microfluidic chip as the printer moves. We define DOA_{CN} as the percentage of correct droplet sequences in the output of the nozzle compared to the droplet sequences generated internally in the microfluidic chip. Meanwhile, DOA_{NM} is defined as the percentage of the correct droplet sequences in the output of the nozzle compared to the deposited sequences in the mold. DOA_{CN} accounts for droplet errors caused by incorrect sequences resulting from deformation in the siphoning area, droplet fusion due to unstable lipid bilayer formation, or droplet rearrangement when the sequence enters the larger nozzle tubing, especially in zig-zag configurations. Similarly, DOA_{NM} considers droplet rearrangement or coalescence during deposition. For both metrics, DOA is calculated as the ratio of correctly sequenced and non-fused droplets to the total number of droplets printed. Fused droplets are counted as the total number of droplets that have coalesced (i.e., if N droplets fuse, there are N errors). The formula for determining DOA is as follows:

$$DOA\% = \frac{Error\ Free\ Droplets}{Total\ Droplets\ Printed} \quad (S4)$$

In the current work, DOA_{CN} is 81.1 ± 17.0 % (Table S1) for three microfluidic chips in which 500-600 droplets were analyzed per chip for a couple off-on siphoning cycles. Figure S9 illustrates a trace for of the DOA_{CN} of one the chips analyzed for two off-on siphoning cycles. We attribute most of the errors in the droplet output accuracy to droplet fusion and sequence rearrangements that occur from local pressure differences during the off- and on-transition states of the siphoning region and sequence rearragnement in the

coupler from the microfluidic chip to the outlet tubing. Sequence stabilization usually takes about 45 to 60 seconds to complete.

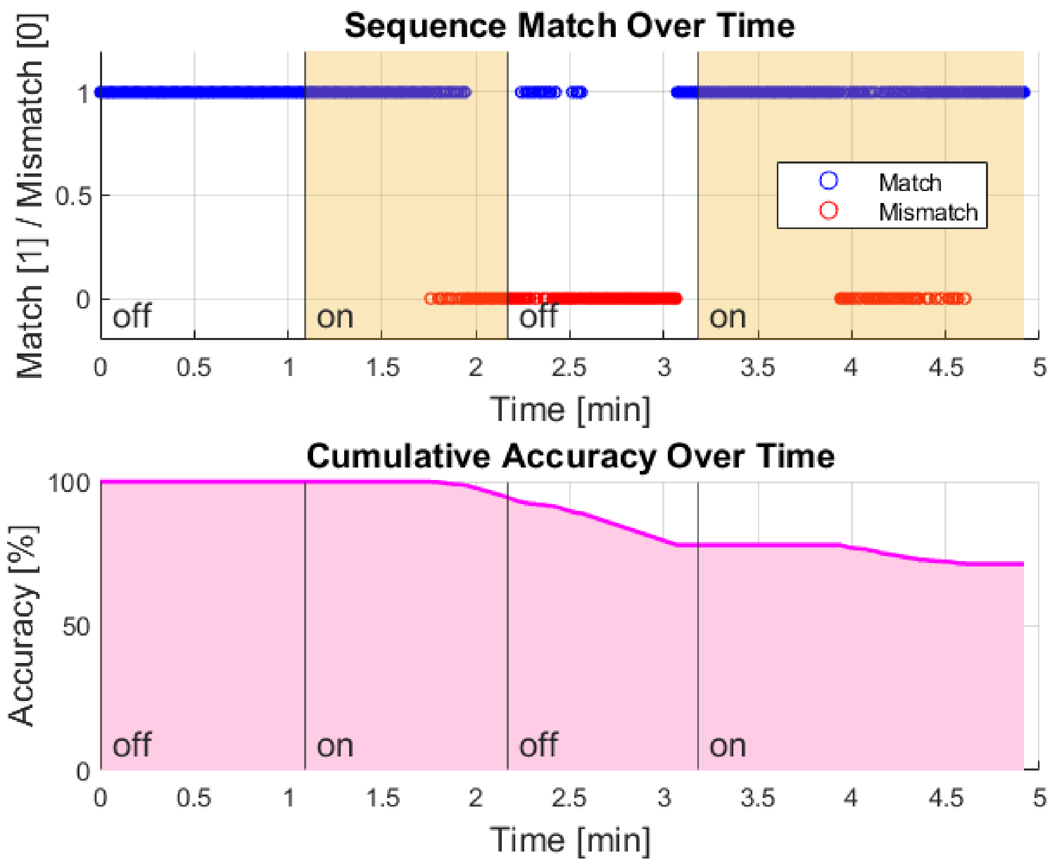

Figure S9: DOA_{CN} for zig-zag thread configuration over time for one chip. The top plot illustrates whether the sequence of droplets generated in the microfluidic chip matches the output sequence at the nozzle during two off-on siphoning cycles. Most errors occur due to pressure changes during these transition states, with sequence stabilization requiring about 45-60 seconds. The bottom plot shows the cumulative accuracy error over time, highlighting the largest accuracy drop occurring during the on-off transition.

DOA_{NM} is 97.5 ± 4.3% across five different prints, each utilizing a new microfluidic chip. To simplify analysis and improve visualization, we use a binary sequence of two inks deposited from the nozzle into a simplified mold. We assess the accuracy of the deposited sequences with Equation S4, where errors include droplet rearrangement after deposition or coalescence during deposition. Figure S10a shows a thread with low DOA_{CN} due to major sequence rearrangements from the chip to the nozzle, but it has a 100% DOA_{NM} as the sequence is successfully deposited into the mold. Figure S10b displays the individual DOA_{NM} for each of the five prints, while Figure S10c zooms in on a segment to demonstrate how a single rearrangement between the chip and nozzle (highlighted in red) can cause a section of the final print to appear random. Based on the results for DOA_{CN} and DOA_{NM}, we conclude that the main source of error in our sequence accuracy stems from the transition between the microfluidic chip and the nozzle tubing. To improve thread accuracy in the final print configurations, additional refinements are needed, such as incorporating valves to enhance control and spacing of droplet sequences.

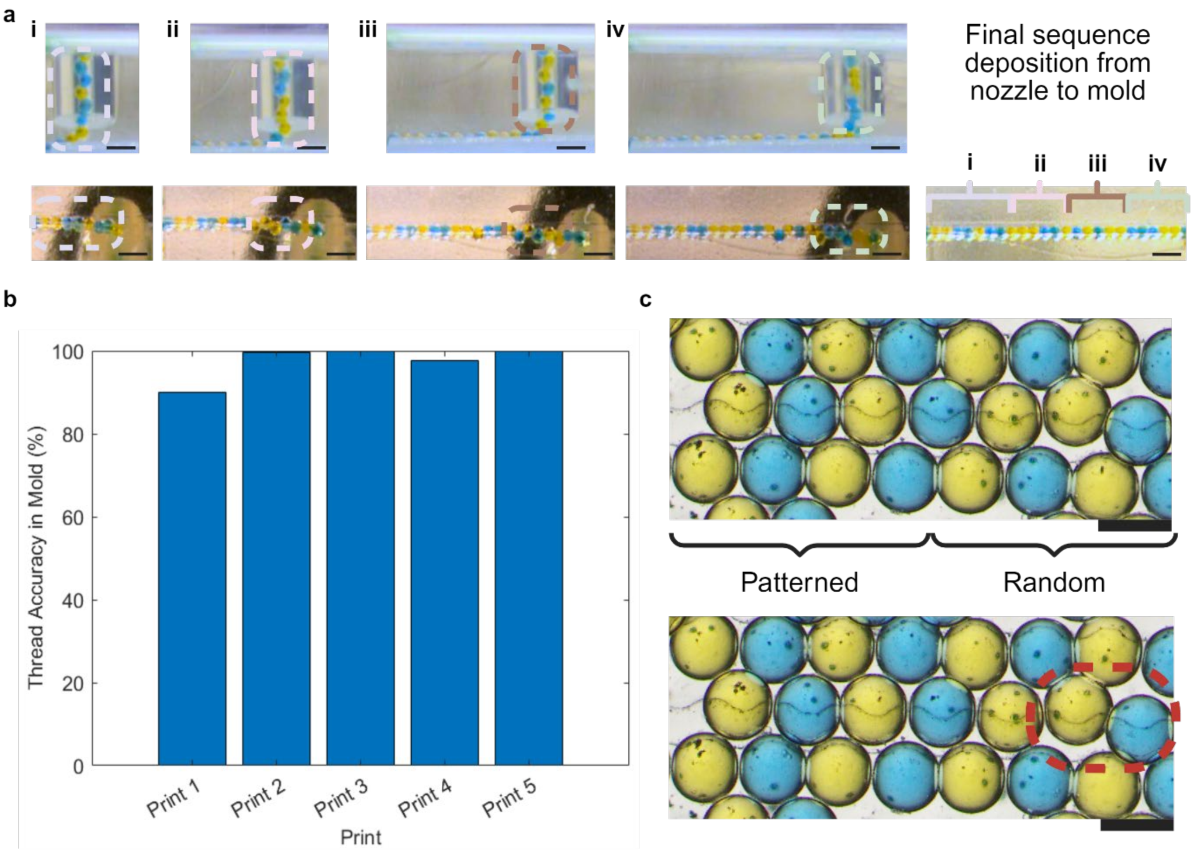

Figure S10: DOA_{NM} is defined as the sequence accuracy of the droplet thread exiting the nozzle compared to its sequence in the mold post deposition. a) Deposition of a droplet sequence with a low DOA_{CN} from the nozzle to the mold. The droplet sequence in the mold accurately matches the deposited sequence from the nozzle (scale bar = 1 mm). b) Bar plots illustrating the DOA_{NM} for five different prints (the total number of droplets analyzed were 120, 237, 198, 220, and 171 for each print 1-5, respectively). The average DOA_{NM} is 97.5 ± 4.3%. c) The top image shows a segment of a binary sequence printed where the left side is patterned while the right side appears random. The bottom image demonstrates that a single error in the middle row produced from droplet rearrangement between the chip and the nozzle is enough to make the appearance appear random (scale bar = 500 µm).

Minimum Feature Size (MFS): For this fabrication technique, the minimum feature size (MFS) of a printed structure is determined by the minimum droplet diameter (MDD) and the packing configuration of the droplets in the print. For simple linear printing (Figure 4C), the minimum feature size is directly the MDD. However, for the zigzag droplet packing configuration (Figure 4D), the MFS increases to effectively match the width of the staggered configuration. Effectively, in this work, the smallest MDD achieves the smallest MFS such that for the linear printing the MFS is 465 µm and for the zigzag configuration, in which the MDD equals the inner tubing diameter of the nozzle, the MFS is approximately 780 µm.

Nozzle Geometry: To reduce the risk of droplet fusion, we use a cylindrical nozzle geometry for this study. Other geometries, such as conical or tapered ones, used in extrusion FDM printing, would impose additional shear forces on the droplet thread that increase the probability of disrupting the lipid bilayers during extrusion [10-11].

XY Resolution: The XY resolution of the print is determined by the accuracy of the stepper motors in locating the nozzle above a particular point on the print bed. Using the

KDC101 motor controllers (Experimental section 4.5), the best XY resolution attainable is 0.05 mm. However, based on our droplet size, we set this resolution to be 0.5 mm.

Layer Height Resolution: The layer height for prints using this system is once again limited by the step resolution of the stepper motors. Therefore, the theoretical best resolution (0.05 mm) and the resolution used in practice (0.5 mm) are the same as the XY resolution.

Hydrogel Viscosity: In conventional hydrogel extrusion printers, successful printing requires a viscosity in the range of 6-30e7 mPa·s or sacrificial bioinks and support baths for inks with lower viscosities [9, 12-13]. However, in the printer described here, as the hydrogels are emulsions in an oil ambient, the individual hydrogel viscosity is less relevant, and the combined viscosity of the oil-lipid-hydrogel precursor is most important. While our hydrogel precursors have viscosities similar to water, ~1 mPa·s , the oil ambient (a combination of undecane and silicone oil) has a higher viscosity of ~11 mPa·s. The lower viscosity of our printing techniques requires specific nozzle velocity and acceleration to maintain printing accuracy, as well as molds for structural support (SI section 9).

Nozzle Velocity and Acceleration: The nozzle velocity and acceleration determine print speed and are limited by the KDC101 motor controllers. These values are manually set constant to 2.3 mm s^{-1} and 1.5 mm s^{-2}, respectively, to ensure a consistent print based on our hydrogel viscosity.

Table S1: Summary of key metrics regarding printing resolution of multi-material hydrogel thread.

**Metric**	**Value**
Minimum droplet size	Theoretical – >100 nm<br>Practical (this work) – 465 ± 15 µm
Chip to nozzle droplet output accuracy (DOA_{CN})	81.1 ± 17.0 %
Nozzle to mold droplet output accuracy (DOA_{NM})	97.5 ± 4.3 %
Minimum feature size (MFS)	Single thread – 465 µm<br>Zigzag thread configuration – 780 µm
Set XY resolution	0.5 mm
Set layer height resolution	0.5 mm
Nozzle velocity	2.3 mm s^{-1}
Nozzle acceleration	1.5 mm s^{-2}

S8. Mechanical properties of polymerized hydrogel thread

To evaluate the interfacial adhesion of the polymerized hydrogel thread, we obtained stress-strain curves and fitted the elastic region, Figures S10 and S11, to estimate the stiffness range of our hydrogel thread to be between 0.2 and 1.82 kPa. The notable experimental differences in hydrogel stiffnesses are well-documented in literature, typically ranging from 0.1 to 300 kPa, due to intra-specimen and inter-specimen variabilities such as hydrogel composition, uneven polymerization, gel thickness, gelation time, hydrogel age, and applied testing method [4-5]. Our hydrogel thread is composed primarily of 5% (W/V) acrylamide monomer (Experimental section 4.2), and our estimated stiffness range (0.2-1.82 kPa) lies within the lower stiffness range (0.1 to 5 kPa) typically reported for 5% (W/V) acrylamide [4-7]. Additionally, acrylamide hydrogels are formulated to behave almost perfectly elastic, as observed in our stress-strain curve in Figure S10, but we observe some plasticity in Figure S11, likely due to uneven polymerization resulting from the hydrogel thread's packing configuration. However, based on the stiffness range for our hydrogel threads, we conclude that the interfacial adhesion between droplets maintains the robustness of the final polymerized hydrogel print.

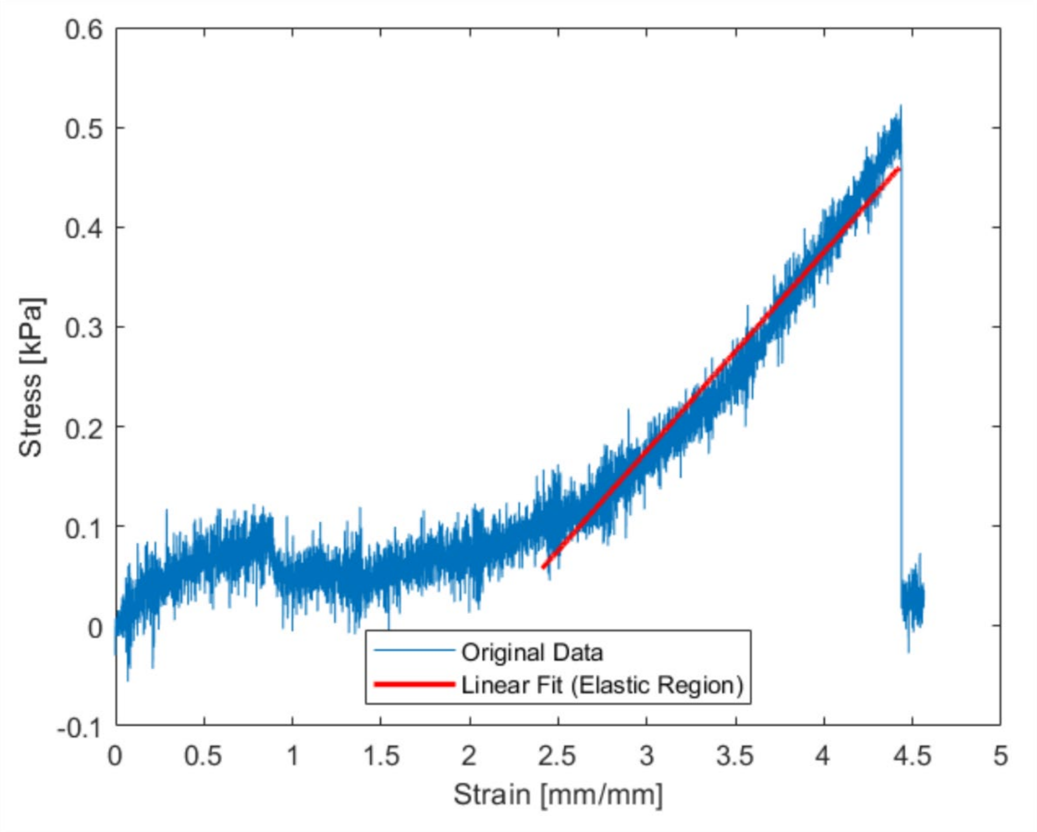

Figure S11: Stress-strain curve of a polymerized hydrogel thread. The elastic region was fitted with a linear model to estimate a Young's modulus of 0.2 kPa. For the initial strain from 0 mm/mm to around 2 mm/mm, the hydrogel sample straightened out as the machine extended, resulting in a very gradual increase in stress.

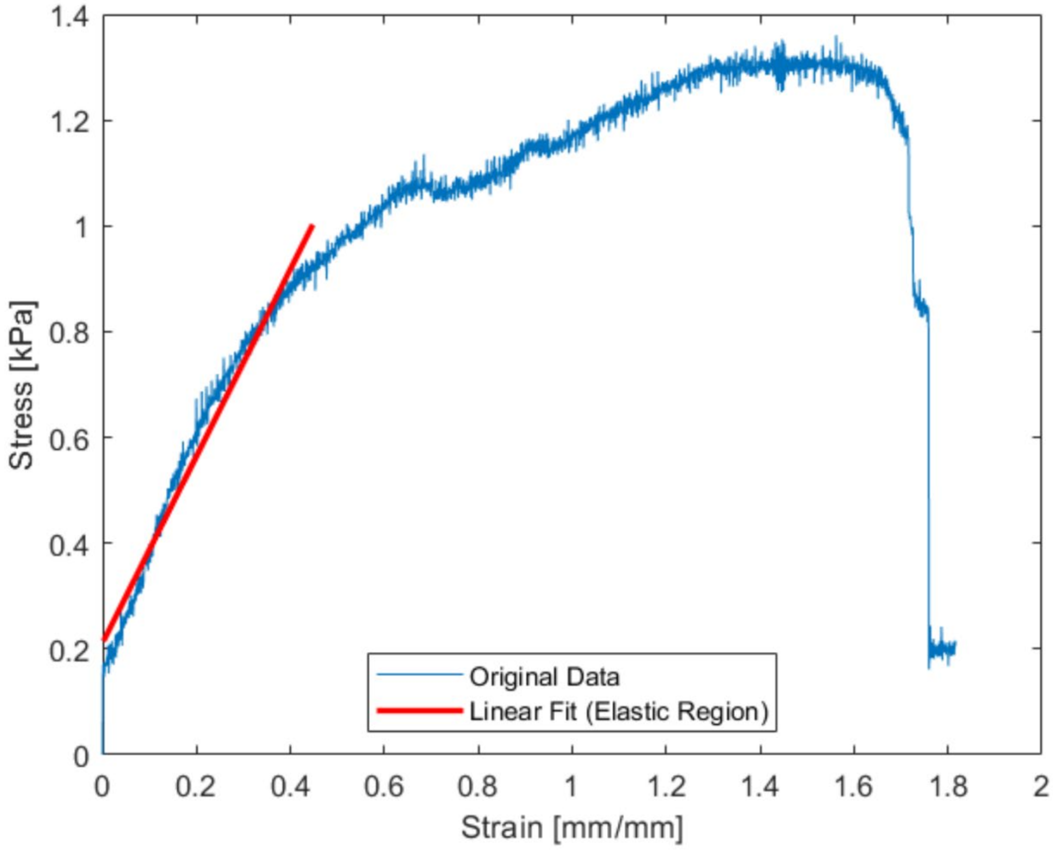

Figure S12: Stress-strain curve of a polymerized hydrogel thread. The elastic region was fitted with a linear model to estimate a Young's modulus of 1.82 kPa. Some plasticity is observed before failure, most likely attributed to an increase in hydrogel polymerization for this sample.

S9. Printing 3D structures

Due to the low viscosity of our pre-polymerized hydrogel solution (as discussed in SI section S7), a mold is necessary to print complex shapes. Without molds, the pre-polymerized hydrogel thread tends to migrate and form amorphous shapes because of movement in the oil environment caused by the nozzle depositing subsequent layers. The dependance on molds for our system is a major drawback, although it has been required for previous formation of large and complex DIB structures [14, 16-19], it is not always required [15]. However, this is a common requirement in extrusion printing with low vidscosity inks [27-29].

To showcase multi-layer 3D printing, we extruded the thread through multiple printing cycles at increasing layer heights until the mold was filled. The figure below shows the filled mold before polymerization (top right) and after polymerization (top left), as well as a comparison of an empty and filled mold to illustrate the depth (bottom). To expand this technique's potential for more complex 3D structures requires exploring alternative supports such as dissolvable supporting baths or partial pre-polymerization of the thread prior to deposition, similar to conventional hydrogel extrusion printing methods [12-13, 25-26].

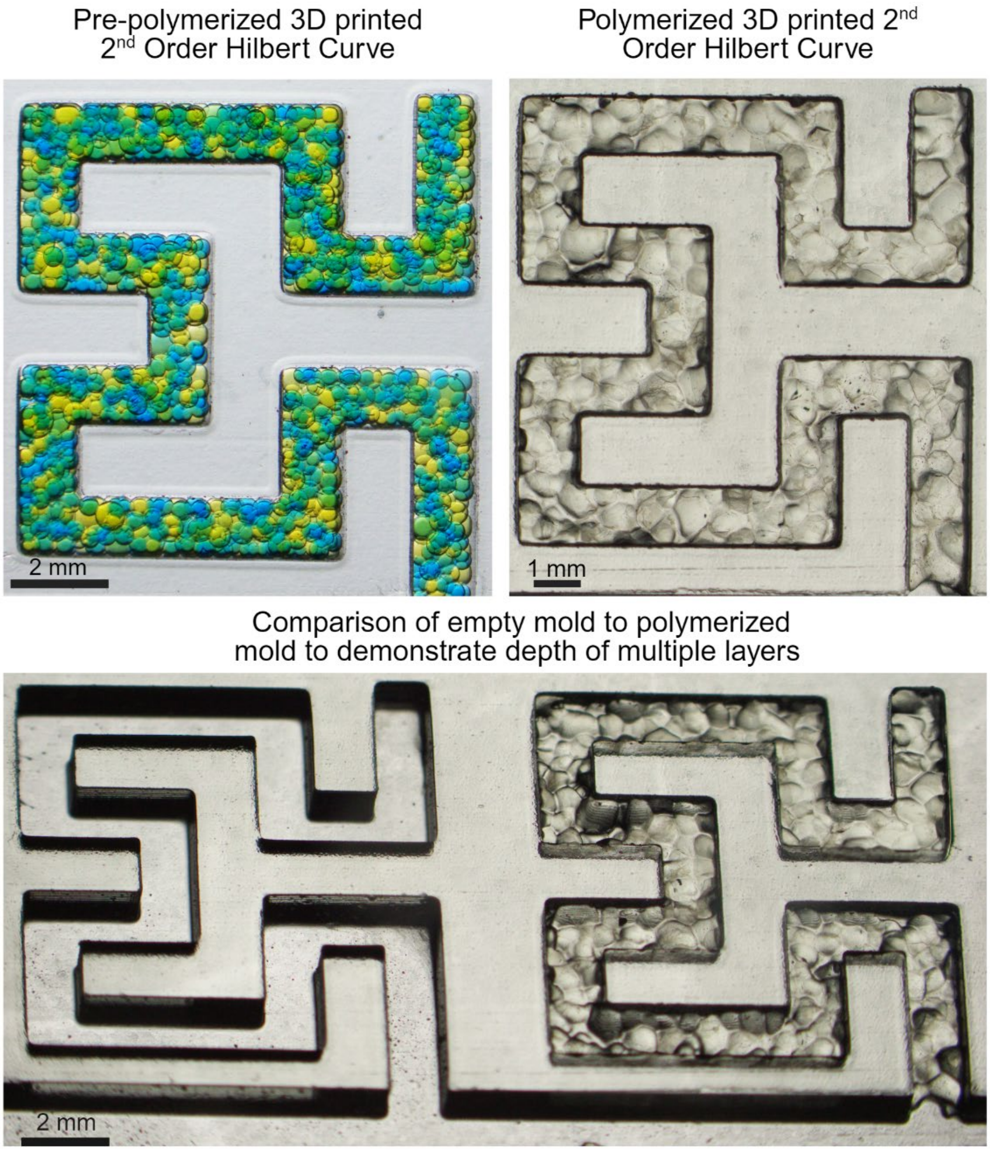

Figure S13: 3D printing of the hydrogel droplet thread still requires a mold. The top images show the mold filled with hydrogel layers, pre-polymerization (left) and post-polymerization (right). The bottom image compares an empty mold to a filled polymerized mold.

S10. Solenoid valve diagram

The siphoning region, which is controlled by on-off states, is regulated by a solenoid valve attached to the outlet tubing of the siphoning channel. Represented here is the electric diagram to control the solenoid valve. The solenoid valve is connected to 12 V power supply which is controlled by a relay module and an Arduino Uno. Upon receiving a user signal, the Arduino switches the relay module to a 12 V supply mode, enabling the solenoid valve to turn on. At the next signal, the relay module switches to its initial state in which no potential reaches the solenoid valve, and it turns off.

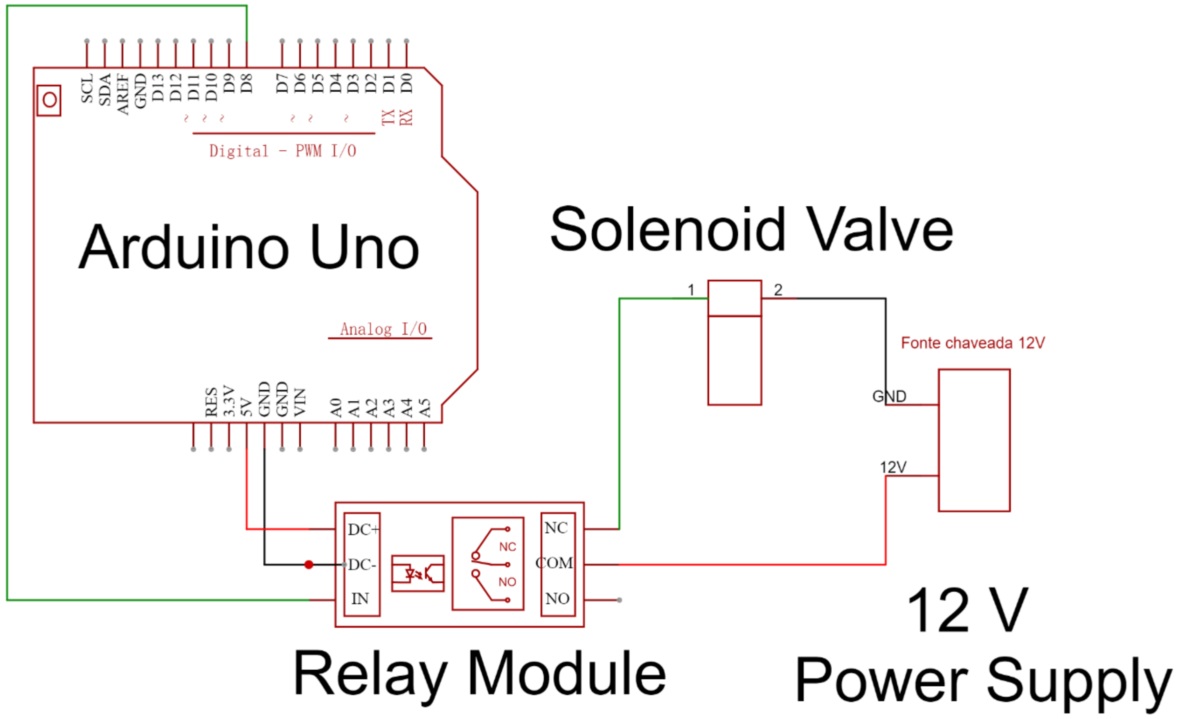

Figure S14: Electric diagram of the hardware to control the solenoid valve for switching between the off and on state of the oil siphoning region.

S11. Experimental setup

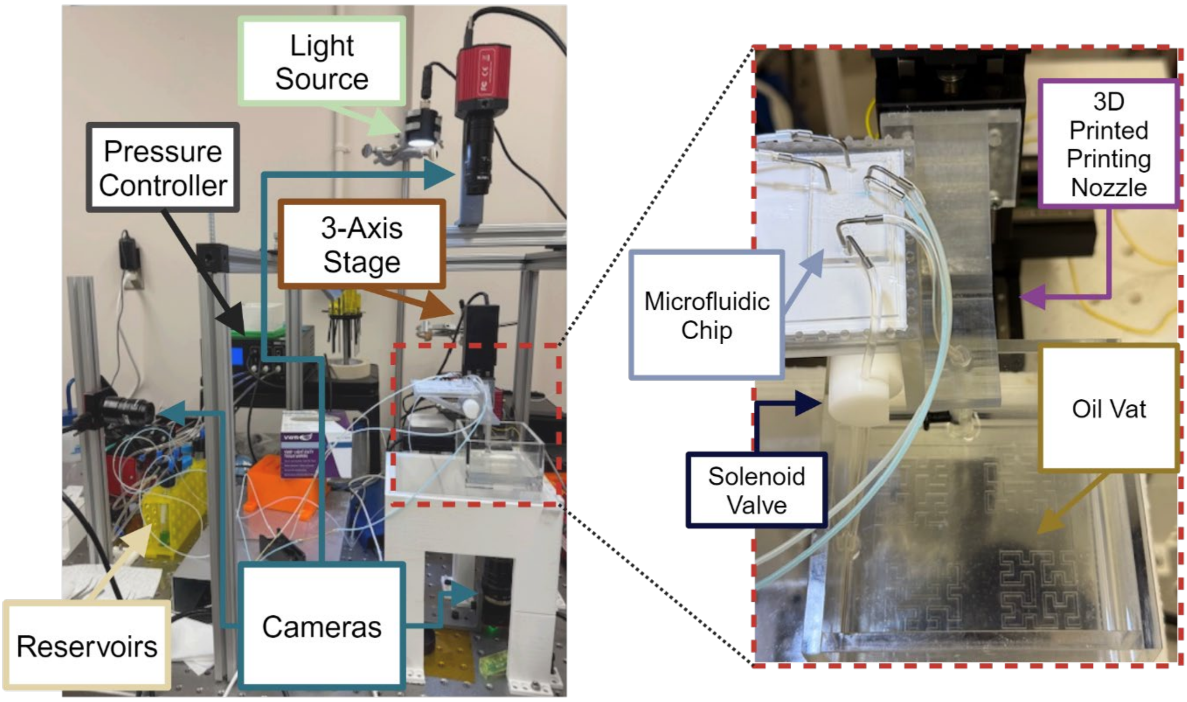

Figure S15: Experimental setup and equipment used for continuous multi-material droplet printing through microfluidic oil siphoning.

References

[1] Bruus, H. (2007). Theoretical microfluidics, volume 18. Oxford university press.

[2] Bruus, H. (2014a). Governing equations in microfluidics. In Microscale Acoustofluidics. The Royal Society of Chemistry.

[3] Ward, K. and Fan, Z. H. (2015). Mixing in microfluidic devices and enhancement methods. Journal of Micromechanics and Microengineering, 25(9):094001.

[4] Milos, F. and del Campo, A. (2024). Polyacrylamide hydrogels as versatile biomimetic platforms to study cell-materials interactions. Advanced Materials Interfaces, 11(34):2400404.

[5] Sheth, S., Jain, E., Karadaghy, A., Syed, S., Stevenson, H., and Zustiak, S. P. (2017). Uv dose governs uv-polymerized polyacrylamide hydrogel modulus. International Journal of Polymer Science, 2017(1):5147482.

[6] Subramani, R., Izquierdo-Alvarez, A., Bhattacharya, P., Meerts, M., Moldenaers, P., Ramon, H., and Van Oosterwyck, H. (2020). The influence of swelling on elastic properties of polyacrylamide hydrogels. Frontiers in Materials, 7:212.

[7] Mann, C. and Leckband, D. (2010). Measuring traction forces in long-term cell cultures. Cellular and Molecular Bioengineering, 3(1):40–49.

[8] Blecua, P., Lipowsky, R., & Kierfeld, J. (2006). Line tension effects for liquid droplets on circular surface domains. *Langmuir*, *22*(26), 11041-11059.

[9] Puza, F. and Lienkamp, K. (2022). 3d printing of polymer hydrogels—from basic techniques to programmable actuation. Advanced Functional Materials, 32(39):2205345.

[10] Chand, R., Muhire, B. S., and Vijayavenkataraman, S. (2022). Computational fluid dynamics assessment of the effect of bioprinting parameters in extrusion bioprinting. International Journal of Bioprinting, 8(2):545.

[11] Reina-Romo, E., Mandal, S., Amorim, P., Bloemen, V., Ferraris, E., and Geris, L. (2021). Towards the experimentally-informed in silico nozzle design optimization for extrusion-based bioprinting of shear-thinning hydrogels. Frontiers in bioengineering and biotechnology, 9:701778.

[12] Brunel, L. G., Hull, S. M., and Heilshorn, S. C. (2022). Engineered assistive materials for 3d bioprinting: support baths and sacrificial inks. Biofabrication, 14(3):032001.

[13] Liu, S., Wang, T., Li, S., and Wang, X. (2022). Application status of sacrificial biomaterials in 3d bioprinting. Polymers, 14(11):2182.

[14] Graham, A. D., Olof, S. N., Burke, M. J., Armstrong, J. P., Mikhailova, E. A., Nicholson, J. G., Box, S. J., Szele, F. G., Perriman, A. W., and Bayley, H. (2017). High-resolution patterned cellular constructs by droplet-based 3d printing. Scientific reports, 7(1):7004.

[15] Jin, Y., Mikhailova, E., Lei, M., Cowley, S. A., Sun, T., Yang, X., Zhang, Y., Liu, K., Catarino da Silva, D., Campos Soares, L., et al. (2023). Integration of 3d-printed cerebral cortical tissue into an ex vivo lesioned brain slice. Nature Communications, 14(1):5986.

[16] Alcinesio, A., Meacock, O. J., Allan, R. G., Monico, C., Restrepo Schild, V., Cazimoglu, I., Cornall, M. T., Krishna Kumar, R., and Bayley, H. (2020). Controlled packing and single-droplet resolution of 3d-printed functional synthetic tissues. Nature communications, 11(1):2105.

[17] Chong, Z., Zeng, Y., Kang, Y., Ding, K., Du, X., and Gu, Z. (2025). Advances in networking droplets. Droplet, 4(2):e173.

[18] Zhang, Y., Tan, C. M., Toepfer, C. N., Lu, X., and Bayley, H. (2024). Microscale droplet assembly enables biocompatible multifunctional modular iontronics. Science, 386(6725):1024–1030.

[19] Zhou, L., Ruiz-Puig, C., Jacobs, B.-A., Han, X., Lisle, R., Bayley, H., and Lu, X. (2021). Bioengineered gastrointestinal tissues with fibroblast-induced shapes. Advanced Functional Materials, 31(6):2007514.

[20] Zhang, Y., Sun, T., Yang, X., Zhou, L., Tan, C. M., Lei, M., and Bayley, H. (2024a). A microscale soft lithium-ion battery for tissue stimulation. Nature chemical engineering, 1(11):691–701.

[21] Downs, F. G., Lunn, D. J., Booth, M. J., Sauer, J. B., Ramsay, W. J., Klemperer, R. G., Hawker, C. J., and Bayley, H. (2020). Multi-responsive hydrogel structures from patterned droplet networks. Nature chemistry, 12(4):363–371.

[22] Venkatesan, G. A., Lee, J., Farimani, A. B., Heiranian, M., Collier, C. P., Aluru, N. R., and Sarles, S. A. (2015). Adsorption kinetics dictate monolayer self-assembly for both lipid-in and lipid-out approaches to droplet interface bilayer formation. Langmuir, 31(47):12883–12893.

[23] Loizou, K., Wong, V.-L., and Hewakandamby, B. (2018). Examining the effect of flow rate ratio on droplet generation and regime transition in a microfluidic t-junction at constant capillary numbers. Inventions, 3(3):54.

[24] Srikanth, S., Raut, S., Dubey, S. K., Ishii, I., Javed, A., and Goel, S. (2021). Experimental studies on droplet characteristics in a microfluidic flow focusing droplet generator: effect of continuous phase on droplet encapsulation. The European Physical Journal E, 44(8):108.

[25] Meng, Z.-J., Liu, J., Yu, Z., Zhou, H., Deng, X., Abell, C., and Scherman, O. A. (2020). Viscoelastic hydrogel microfibers exploiting cucurbit [8] uril host–guest chemistry and microfluidics. ACS Applied Materials & Interfaces, 12(15):17929–17935.

[26] Qiu, J., Ma, S., and Qu, X. (2025). Challenges and innovative strategies in 3d printing of natural biomolecular hydrogels. Nano Select, 6(6):e202400149.

[27] Budharaju, H., Sundaramurthi, D., & Sethuraman, S. (2024). Embedded 3D bioprinting–An emerging strategy to fabricate biomimetic & large vascularized tissue constructs. *Bioactive Materials*, *32*, 356-384.

[28] Compaan, A. M., Song, K., & Huang, Y. (2019). Gellan fluid gel as a versatile support bath material for fluid extrusion bioprinting. *ACS applied materials & interfaces*, *11*(6), 5714-5726.

[29] Hinton, T. J., Jallerat, Q., Palchesko, R. N., Park, J. H., Grodzicki, M. S., Shue, H. J., ... & Feinberg, A. W. (2015). Three-dimensional printing of complex biological structures by freeform reversible embedding of suspended hydrogels. *Science advances*, *1*(9), e1500758.

[30] Souto, E. B., Cano, A., Martins-Gomes, C., Coutinho, T. E., Zielińska, A., & Silva, A. M. (2022). Microemulsions and nanoemulsions in skin drug delivery. *Bioengineering*, *9*(4), 158.

[31] Rosen, M.J. and Kunjappu, J.T. (2012). Emulsification by Surfactants. In Surfactants and Interfacial Phenomena (eds M.J. Rosen and J.T. Kunjappu). https://doi.org/10.1002/9781118228920.ch8

[32] McClements, D. J. (2012). Nanoemulsions versus microemulsions: terminology, differences, and similarities. *Soft matter*, *8*(6), 1719-1729.
